# Prokaryotic bias in surface ocean particles

**DOI:** 10.1073/pnas.2500624123

**Published:** 2026-04-01

**Authors:** Yeongjun Ryu, Ashley E. Maloney, Victoria H. Luu, Lingkun Guo, Sergey Oleynik, Sarah E. Fawcett, Meytal B. Higgins, Nicolas Van Oostende, Bess B. Ward, Claire C. Z. Cook, Natalie R. Cohen, Erica Ewton, Susanne Menden-Deuer, Julie Granger, Adrian Marchetti, Hedy M. Aardema, Hans A. Slagter, Ralf Schiebel, Alfredo Martínez-García, Gerald H. Haug, Daniel M. Sigman

**Affiliations:** ^a^Department of Geosciences, Princeton University, Princeton, NJ 08544; ^b^Climate Geochemistry Department, Max Planck Institute for Chemistry, Mainz 55128, Germany; ^c^Department of Geological Sciences, University of Colorado, Boulder, CO 80309; ^d^Department of Oceanography, University of Cape Town, Rondebosch 7701, South Africa; ^e^Marine and Antarctic Research Centre for Innovation and Sustainability, University of Cape Town, Rondebosch 7701, South Africa; ^f^ExxonMobil Technology and Engineering Company, Annandale, NJ 08801; ^g^Skidaway Institute of Oceanography, University of Georgia, Savannah, GA 31411; ^h^Graduate School of Oceanography, University of Rhode Island, Narragansett, RI 02882; ^i^Department of Marine Sciences, University of Connecticut, Groton, CT 06340; ^j^Department of Earth, Marine and Environmental Sciences, University of North Carolina, Chapel Hill, NC 27514; ^k^Department of Earth and Planetary Sciences, Zürich 8092 ETH, Switzerland

**Keywords:** particulate organic matter, phytoplankton, nitrogen isotopes, chlorophyll

## Abstract

While the ocean’s photosynthetic production of organic matter rivals that on land, a combination of heterotrophy and sinking prevents significant accumulation of particulate organic matter (POM) in open ocean surface waters. The origins and fates of POM in ocean surface waters are unclear, in part due to the dominance of nonliving, altered material. From the natural nitrogen isotopic composition of chlorophyll and its degradation products, we estimate the fraction of particles from eukaryotic vs. prokaryotic phytoplankton. In subtropical gyres and along the eastern North Pacific margin, the eukaryotic-to-prokaryotic ratio in particles matches that of living phytoplankton. However, in the North Atlantic outside its subtropical gyre, particles have a lower eukaryotic-to-prokaryotic ratio than do the living phytoplankton. This discrepancy at least partly arises from preferential sinking of eukaryotic biomass, consistent with the canonical but disputed paradigm that cyanobacteria disproportionately fulfill the energetic demands of the upper ocean microbial community while eukaryotes drive export production. The prokaryotic bias in surface ocean particles may also result from slow decomposition of specific components of prokaryotic biomass, a possible bottleneck in the ocean’s microbial loop. The different fates of organic matter produced by eukaryotic and prokaryotic phytoplankton affect the productivity of the surface ocean, carbon export to the interior, and the signals recorded in deep-sea sediments.

## Significance

Eukaryotic and prokaryotic phytoplankton are the ultimate sources of most organic matter in the ocean, yet there is uncertainty in their relative contributions to surface ocean carbon stocks and their export to depth. Here, the nitrogen isotopic composition of chlorophyll and its degradation products is used to quantify relative contributions of eukaryotic versus prokaryotic phytoplankton to dead particulate organic matter in the surface ocean. Compared to the eukaryotic-to-prokaryotic ratio of living phytoplankton, we observe preferential retention of prokaryotic biomass in surface particles. This ‘prokaryotic bias’ results from preferential sinking of eukaryotic material and perhaps also slow decomposition of some prokaryote-derived biomolecules. This bias impacts upper ocean carbon stocks, carbon export, and the biological signals recorded in deep-sea sediments.

Upper ocean ecosystems rely on the energy and carbon generated through the photosynthetic production of organic matter by phytoplankton (1). The sinking export of a fraction of this organic matter out of the sunlit surface sequesters CO_2_ in the ocean interior, lowering the atmospheric concentration of CO_2_ (2). Together, *Prochlorococcus* and *Synechococcus* (hereafter “prokaryotes”), along with eukaryotic phytoplankton (hereafter “eukaryotes”), predominantly drive photosynthetic production in the open ocean (3). The composition of the phytoplankton community varies widely, playing a central role in determining the characteristics of the rest of the upper ocean ecosystem (4) and possibly the magnitude and efficiency of carbon export (5).

In seawater, particulate organic matter (POM) is broadly categorized into suspended and sinking particles. Suspended particles comprise most of the standing stock of organic particles in the surface layer, while sinking particles facilitate carbon export to the ocean interior and seabed (6). Particle size has long been considered the primary determinant of a particle’s tendency to be exported (7), as it affects the gravitational sinking rate (8) as well as the likelihood of consumption by the highly efficient microbial loop (9). These concepts have led to the prediction that, compared to large eukaryotic phytoplankton, prokaryotic and small eukaryotic phytoplankton contribute disproportionately less to export production than they do to net primary production (10), suggesting the potentially biased contribution of specific phytoplankton groups to export vs. recycled production. However, this long-held view has been challenged, with arguments that small phytoplankton are efficiently exported by aggregation and other processes (11, 12).

The role of different organisms in recycled and export production can be clarified by characterizing the origin of suspended particles in surface waters. The phytoplankton fractions in the living phytoplankton community have been investigated widely with flow cytometry (13, 14). However, flow cytometry does not characterize nonliving particles, which dominate the total particle pool (15).

**Fig. 1.**
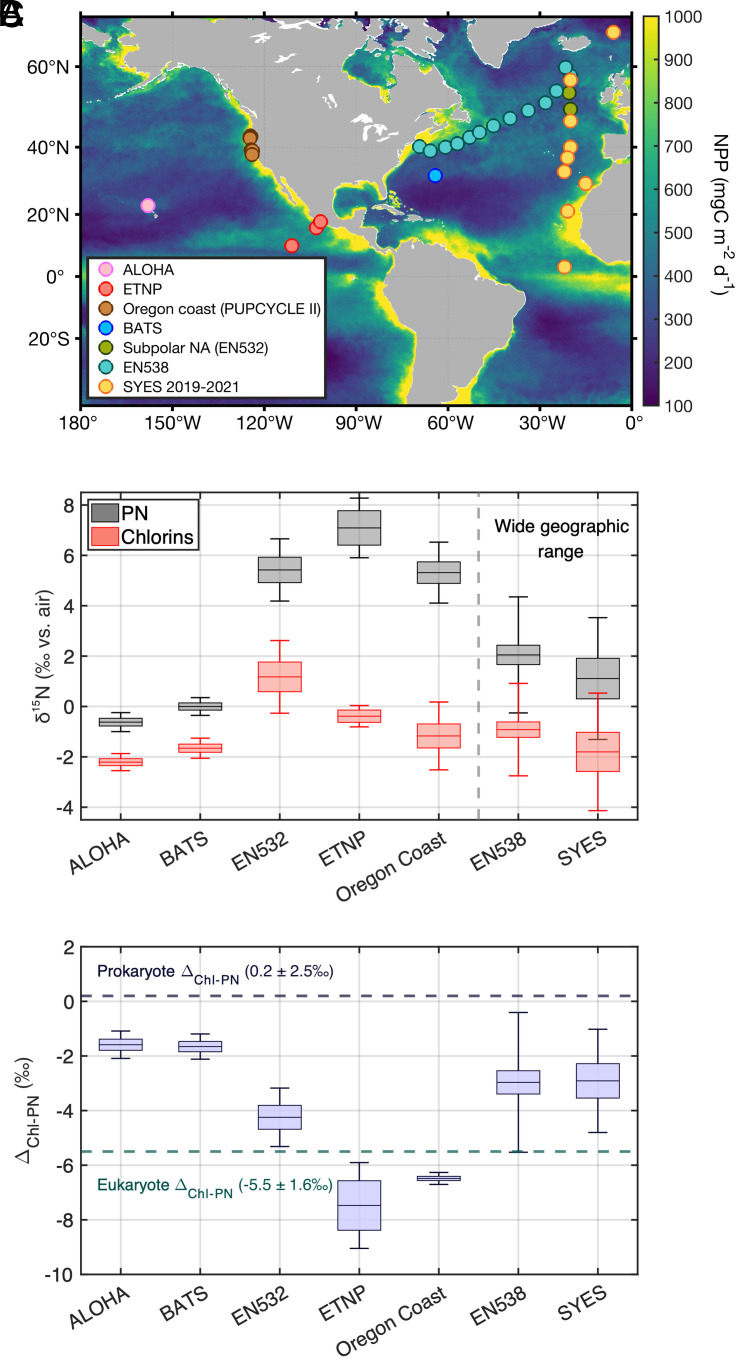
Sampling stations and isotopic measurements of suspended PN and chlorin N. (*A*) Locations of sampling stations plotted over the annual mean net primary production (NPP) estimates for 2022 from the Carbon-based Production Model with Visible Infrared Imaging Radiometer Suite (VIIRS) satellite image (24) (pink: Station ALOHA, red: eastern tropical North Pacific (ETNP), brown: PUPCYCLE II cruise stations off the Oregon coast, blue: Bermuda Atlantic Time-series Study (BATS) Station, olive: EN532 cruise stations, cyan: EN538 cruise stations, yellow: SYES cruise stations). (*B*) Average δ^15^N of bulk suspended PN (gray box), chlorin (red box), and (*C*) their differences (Δ_Chl-PN_; blue box) in the different sampling regions. Green and dark blue dashed lines indicate the two Δ_Chl-biomass_ endmembers (eukaryotes: –5.5 ± 1.6‰, prokaryotes: 0.2 ± 2.5‰). The heights of the box plots and the error bars represent the 1SE and 1SD of measurements.

Laboratory culture studies have documented a difference between prokaryotic and eukaryotic marine phytoplankton in the nitrogen (N) isotopic discrimination associated with chlorophyll biosynthesis (16–18). Here, we use this difference to estimate the ratio of eukaryote-to-prokaryote-derived organic matter in the suspended particles of North Atlantic and North Pacific surface waters, based on N isotopic measurements of their chlorophyll and chlorophyll degradation products [hereafter collectively referred to as “chlorins (19)”]. The samples were collected from various oceanic regimes, including subtropical gyres in both North Atlantic and North Pacific basins, the coastal upwelling zone near the Oregon coast, the eastern tropical North Pacific (ETNP), and a range of nongyre environments in the North Atlantic (20) (Fig. 1*A*). These estimates are compared with independent measures of eukaryotic vs. prokaryotic phytoplankton biomass contributions from flow cytometry cell-count data (21–23). In addition, from a subset of stations, the same isotopic analyses were conducted on particles from below the euphotic zone, to characterize the effects of i) degradation on subsurface particles and ii) vertical mixing on surface particles.

## Results

Culture studies report that the δ^15^N difference between the chlorophyll of phytoplankton (δ^15^N_Chl_) and their bulk biomass (δ^15^N_biomass_) is distinct between eukaryotes and prokaryotes (16–18) [δ^15^N = 1,000 * ((^15^N/^14^N)_sample_/(^15^N/^14^N)_air N2_ – 1), in ‰ (permil, or parts per thousand) relative to atmospheric N_2_]. The studies find that eukaryotic phytoplankton produce chlorophyll that is 5.5 ± 1.6‰ lower in δ^15^N than their biomass (Δ_Chl-biomass_ = −5.5 ± 1.6‰, where Δ_Chl-biomass_ = δ^15^N_Chl_ – δ^15^N_biomass_), whereas marine cyanobacteria show almost no N isotopic difference between chlorophyll and biomass (Δ_Chl-biomass_ = 0.2 ± 2.5‰). Here, in suspended particles collected from ocean surface waters (i.e., within the mixed layer), δ^15^N_Chl_ is determined for the chlorin compound class, encompassing both chlorophyll and phaeopigments, to include degraded chlorophyll. δ^15^N of suspended particulate nitrogen (PN) (δ^15^N_PN_) is also analyzed in the same filtered particles and compared with δ^15^N_Chl_.

Δ_Chl-PN_ (Δ_Chl-PN_ = δ^15^N_Chl_ – δ^15^N_PN_) varies across surface ocean environments (Fig. 1*C*), mainly due to δ^15^N_PN_, with the δ^15^N_Chl_ varying less (Fig. 1*B* and *SI Appendix,* Fig. S1). The Δ_Chl-PN_ values from the subtropical gyre sites, Station ALOHA in the North Pacific and the Bermuda Atlantic Time-series Study (BATS) site in the North Atlantic, are −1.6 ± 0.5‰ (*n* = 6) and −1.7 ± 0.5‰ (*n* = 6), respectively. In contrast, the ETNP and Oregon coast show more strongly negative Δ_Chl-PN_ values [i.e., a higher magnitude of chlorophyll ^15^N depletion relative to bulk particles; −7.5 ± 1.6‰ (*n* = 3) and −6.5 ± 0.2‰ (*n* = 8), respectively]. At the subpolar North Atlantic stations sampled during the late summer (two subpolar stations from the EN532 cruise), Δ_Chl-PN_ values are higher (−4.2 ± 1.1‰, *n* = 6). The EN538 cruise and the SYES (S/Y *Eugen Seibold*) samples cover, respectively, a broad range of environments from the temperate to subpolar western North Atlantic and from the eastern tropical to subpolar North Atlantic. Accordingly, their Δ_Chl-PN_ ranges are also broad (EN538: −6.7 to 2.0‰; SYES: –6.0 to −0.2‰; *SI Appendix,* Fig. S1). The subpolar North Atlantic samples of EN538 (which were collected in early spring) and of SYES samples (collected in summer) are both relatively high [−2.5 ± 2.2‰ (*n* = 14) and −3.7 ± 0.2‰ (*n* = 2)]. The subtropical to tropical samples from SYES are even higher [−1.2 ± 1.2‰ (*n* = 4)] (*SI Appendix,* Fig. S2).

**Fig. 2.**
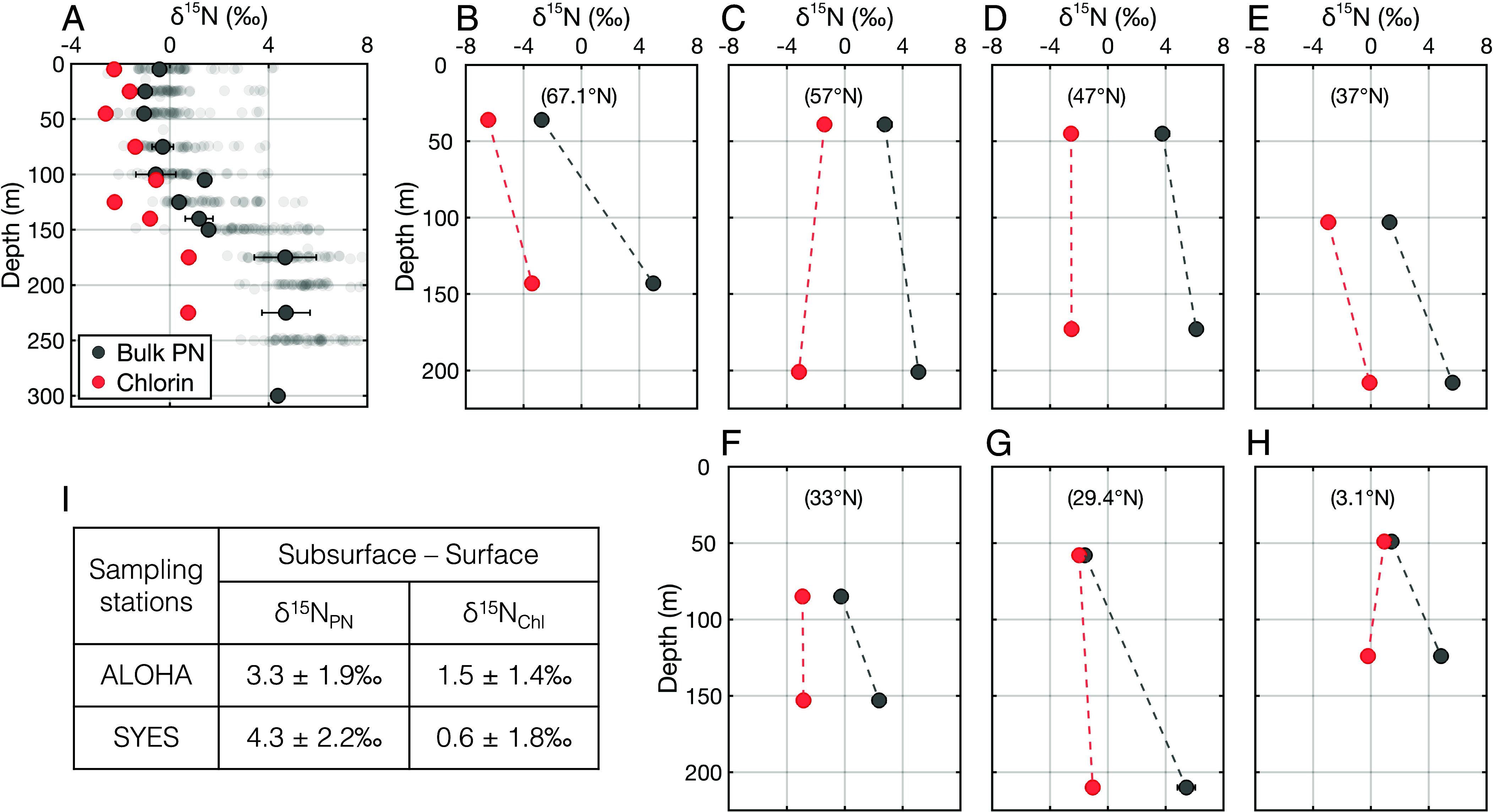
Vertical profiles of δ^15^N for bulk suspended PN (solid gray circles) and chlorin (red circles) at (*A*) Station ALOHA and (*B*–*H*) SYES stations. Changes in δ^15^N from surface (upper 100 m) to subsurface (100–300 m) are shown in (*I*) as differences between subsurface and surface δ^15^N. In (*A*), transparent gray circles indicate bulk PN measurements over time from the Station ALOHA (https://hahana.soest.hawaii.edu/hot/). In (*B*–*H*), the latitudes of the SYES sampling stations are indicated in parentheses. Error bars represent the 1SD of replicate measurements. Bulk PN samples were measured at least twice to assess analytical precision, whereas replicate measurements were not available for chlorins. The analytical precision of bulk PN δ^15^N is typically better than 0.3‰; therefore, error bars are smaller than the symbol size for most data points (gray circles).

At Station ALOHA and the SYES stations, subsurface samples were analyzed to examine the vertical structure of δ^15^N_PN_ and δ^15^N_Chl_ (Fig. 2). At Station ALOHA, δ^15^N_PN_ is relatively uniform in the upper 100 m (−0.7 ± 0.3‰) but increases with depth below the euphotic zone, reaching 4.6 ± 0.2‰ below 175 m. In contrast, δ^15^N_Chl_ exhibits a much smaller change with depth, increasing modestly from −2.0 ± 0.5‰ within the upper 100 m to 0.8 ± 0.1‰ below 175 m.

A similar pattern is observed across the SYES stations. While δ^15^N_PN_ increases by 2.4 to 7.0‰ from the upper 100 m to the subsurface (124–210 m), δ^15^N_Chl_ changes are on average only +0.6‰ over the same depth interval. We cannot rule out that the small surface-to-subsurface increase in δ^15^N_Chl_ reflects weak isotopic fractionation associated with chlorin degradation. However, at a few stations, δ^15^N_Chl_ instead decreases slightly with depth, and a paired *t* test indicates that the δ^15^N_Chl_ increase with depth is not statistically significant (*P* >0.1). The variability in the vertical trend in δ^15^N_Chl_ warrants further investigation; we suspect that it relates to lateral transport and/or the different time scales integrated by surface and subsurface chlorin pools. In any case, at both Station ALOHA and the SYES stations, Δ_Chl-PN_ shifts toward strongly negative values in the subsurface, driven by rising δ^15^N_PN_ and relatively stable δ^15^N_Chl_, a pattern best explained by isotopic fractionation during PN degradation in the subsurface.

## Discussion

### Comparison of Phytoplankton Fraction in Suspended Particles and Living Biomass.

Given the Δ_Chl-biomass_ values of cultured organisms, our observed spatial pattern in Δ_Chl-PN_ is consistent with the previously reported numerical dominance of prokaryotes in the subtropical gyres (21, 22) and eukaryotes in nutrient-rich regions such as the marginal ocean and subpolar regions (25). The δ^15^N of both chlorins and bulk suspended PN reflect their phytoplankton origins, with little subsequent change during degradation in the euphotic zone. While heterotrophic biomass contributes to bulk suspended PN, it does not contribute to the chlorin pool. Moreover, despite the significant heterotrophic bacterial contribution to bulk PN, the δ^15^N of bulk PN remains close to that of phytoplankton (*SI Appendix*). Thus, the difference between δ^15^N_Chl_ and δ^15^N_PN_ is a potential metric for the relative contribution of eukaryotic vs. prokaryotic phytoplankton to suspended particles.

**Fig. 3.**
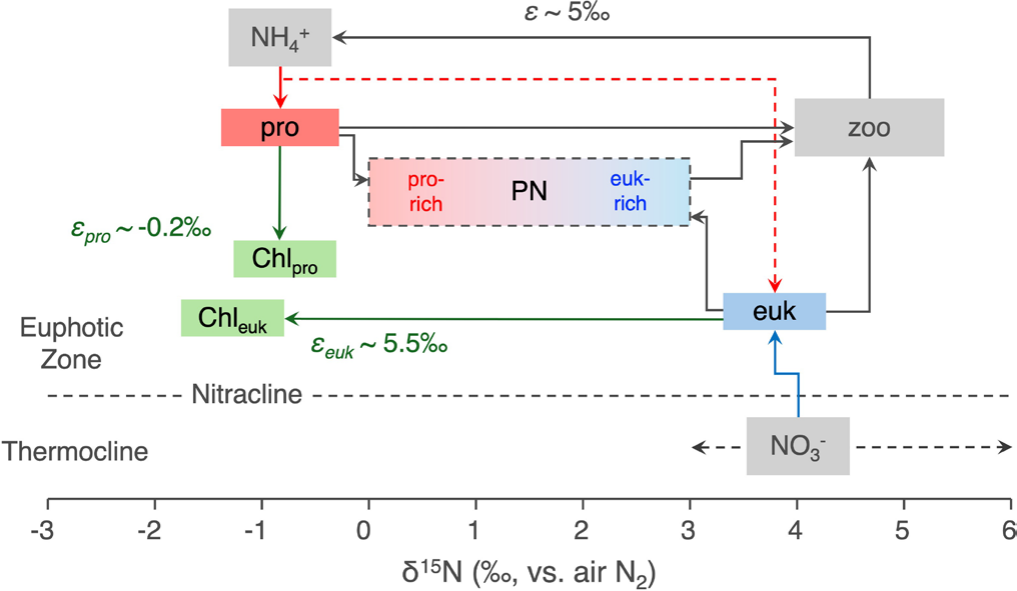
Schematic representation of nitrogen cycling with N isotopic composition. Upward blue arrow depicts the input of subsurface nitrate, which has a δ^15^N of ~3 to 6‰ across the study regions, as indicated by the gray dashed arrows, excluding the ETNP. Red arrows indicate recycled N assimilation with the red dashed arrow emphasizing that eukaryotes derive less N from recycled sources compared to new nitrate. The offset of eukaryotes from nitrate is not due to isotopic fractionation during nitrate assimilation, the consumption of which is complete at most of the study sites, but rather to the additional use of recycled N by eukaryotes. Green arrows represent N isotope fractionation during chlorophyll synthesis in cyanobacteria and eukaryotic phytoplankton. Abbreviations: “pro,” prokaryotic phytoplankton; “euk,” eukaryotic phytoplankton; “zoo,” herbivorous zooplankton; “chl,” chlorophyll; “PN,” particulate nitrogen. The δ^15^N values of eukaryotic phytoplankton and prokaryotic phytoplankton are marked with blue and red boxes, and depending on their relative contribution to the suspended PN pool, its δ^15^N value is determined. Zooplankton are elevated in δ^15^N due to preferential metabolism of low-δ^15^N ammonium, which is effluxed and provides recycled N to the phytoplankton. For simplicity, sinking organic matter is not included in the diagram.

One caveat is that, in comparison to chlorin N, the taxonomic origin of bulk PN may more strongly affect its tendency to be degraded. However, the different N sources to eukaryotic vs. prokaryotic phytoplankton and their different biomass-to-chlorophyll isotopic fractionations conspire to generate chlorophyll δ^15^N values that are similar for the two taxonomic groups. Flow cytometry-sorted biomass δ^15^N measurements in the Sargasso Sea reveal that, due to N source differences, eukaryotic phytoplankton have δ^15^N values that are on average ~5‰ higher than the cyanobacteria *Synechococcus* and *Prochlorococcus* (21). This δ^15^N offset between eukaryotic phytoplankton and cyanobacteria is consistently observed not only in the Sargasso Sea but also in the subpolar North Atlantic (*SI Appendix*). However, eukaryotic phytoplankton produce chlorophyll with a δ^15^N ~5.5‰ lower than their biomass. These two effects offset one another to yield similar δ^15^N_Chl_ values for both cyanobacteria and eukaryotic phytoplankton (Fig. 3), contributing to the relative similarity of δ^15^N_Chl_ among our study sites (Fig. 1*B*). The similarity of eukaryotic and prokaryotic δ^15^N_Chl_ makes the calculation framework below insensitive to possible differences in the degrees to which the taxonomic origins of chlorin N and bulk PN influence their degradation. This also simplifies the Δ_Chl–PN_ mass balance, making it largely insensitive to variations in chlorin content or chlorin-to-carbon ratios, which can be affected both by environmental conditions [e.g., light and nutrient availability (26)] and by the possibility of differential preservation of chlorophyll relative to other nonchlorophyll biomass following cell death (*SI Appendix*).

We use the following equation for the relative fractions of eukaryotic phytoplankton-originating particulate N (f_euk,PN_) and prokaryotic phytoplankton-originating particulate N (f_pro,PN_):

[1] $$\begin{array}{c} {△}_{Chl-PN}=f_{euk,PN}\times {△}_{Chl-biomass,euk}+f_{pro,PN}\\ \times {△}_{Chl-biomass,pro}, \end{array}$$

where Δ_Chl-biomass,euk_ and Δ_Chl-biomass,pro_ are set to the average values from cultured organisms (Δ_Chl-biomass,euk_ = −5.5 ± 1.6‰, Δ_Chl-biomass,pro_ = 0.2 ± 2.5‰) (16–18), and f_pro,PN_ can be substituted with (1 – f_euk,PN_). This yields an expression for f_euk,PN_ in terms of the measured Δ_Chl-PN_ value:

[2] $${△}_{Chl-PN}=f_{euk,PN}\times -5.5‰+[(1-f_{euk,PN})\times 0.2‰].$$

[3] $$f_{euk,PN}=\frac{{△}_{Chl-PN}-0.2‰}{-5.7‰}.$$

**Fig. 4.**
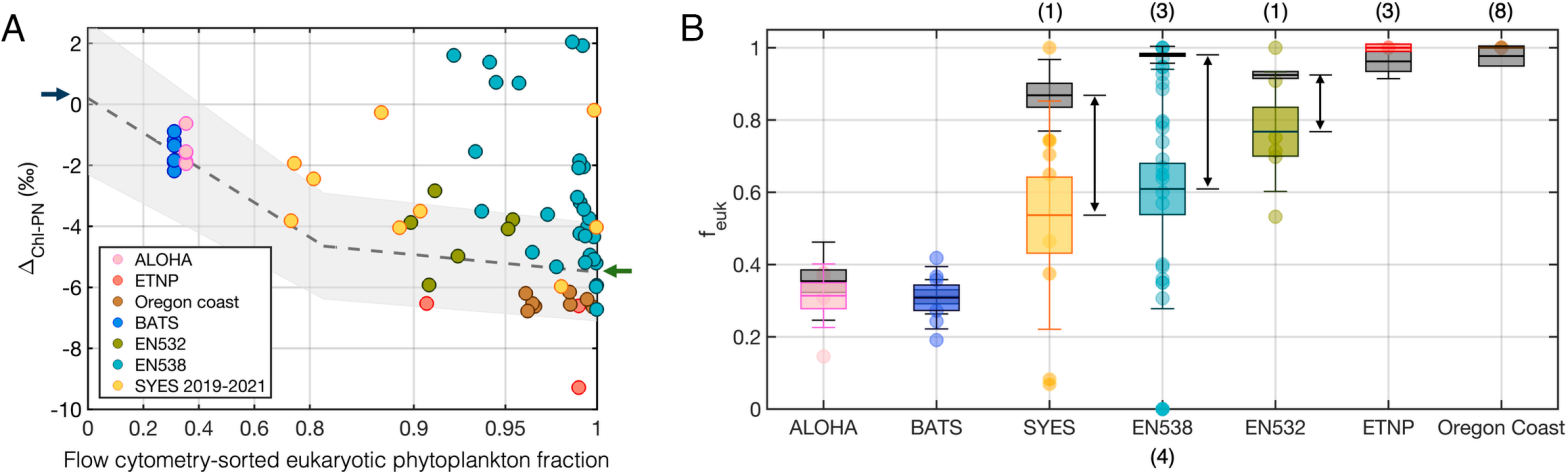
Comparison of calculated eukaryotic fractions in suspended particles and living phytoplankton. (*A*) Δ_Chl-PN_ plotted vs. flow cytometry-sorted eukaryotic phytoplankton fraction (f_euk,phyto_), with stations color-coded (pink: ALOHA, red: ETNP, brown: Oregon coast, blue: BATS, olive: EN532 (subpolar North Atlantic), cyan: EN538 (North Atlantic), yellow: SYES (eastern North Atlantic)). Dashed line with gray shading indicates expected Δ_Chl-PN_ values from the two-endmember mixing model (Eq. **3**). The two Δ_Chl-biomass_ endmembers (prokaryotes: 0.2 ± 2.5‰, eukaryotes: –5.5 ± 1.6‰) are denoted as dark blue and green arrows, respectively. (*B*) Comparison between f_euk,phyto_ (gray box) and f_euk,PN_ (colored box), arranged in order of NPP. Each f_euk,PN_ calculation is represented by a colored circle. Samples with f_euk,PN_ of >1 or <0 were plotted at 1 and 0, with number of cases noted in parentheses on top and bottom axes. The heights of the boxes and error bars represent the 1SE and 1SD, respectively. For the subtropical samples without flow cytometry data from the same cruises, estimates were drawn from previous flow cytometry datasets (21, 22). At the ETNP stations, where flow cytometry data from the same cruise were also unavailable, either nearby offshore flow cytometry data (23) or size-fractionated particle biomass data from marginal sites (28) were used. Black double-headed arrows highlight discrepancies between f_euk,PN_ and f_euk,phyto_ in the North Atlantic. In the ETNP and Oregon coast samples, all Δ_Chl-PN_ values fall below the eukaryotic endmember, yielding calculated f_euk,PN_ values >1; therefore, error bars are not shown because the estimates exceed the bounded 0-1 range of the mixing model.

Thus, lower (more negative) Δ_Chl-PN_ represents a larger (negative) difference between δ^15^N_Chl_ and δ^15^N_PN_, which implies a higher f_euk,PN_. We compare these estimates of the Δ_Chl-PN_-derived eukaryotic fraction (f_euk,PN_) with the fraction of eukaryote cells in the living biomass recovered from flow cytometry cell counts (f_euk,phyto_) (Fig. 4). In this comparison, f_euk,PN_ is based on PN and thus must be converted to organic carbon for comparison to the flow cytometry estimates of phytoplankton f_euk_. If the C:N ratio differs between prokaryotic and eukaryotic phytoplankton, f_euk,PN_ will be different for N and C. However, this effect appears limited, as *Prochlorococcus* has only a slightly lower C:N ratio than eukaryotes while *Synechococcus* has a similar C:N ratio to eukaryotes (27), and we use these literature values when considering f_euk_ in N and C units below. Even where *Prochlorococcus* contributes substantially to biomass, as at the oligotrophic gyre stations, the effect of its different C:N on the calculated f_euk_ is minor.

The relatively high Δ_Chl-PN_ values (i.e., smaller amplitude negative values) observed in the subtropical gyres (ALOHA: −1.6 ± 0.5‰, BATS: −1.7 ± 0.5‰) suggest that prokaryotes contribute substantially to the suspended particles in these oligotrophic regions. Estimated f_euk,PN_ at ALOHA and BATS are approximately 31.4 ± 5.3% and 32.6 ± 4.6%, respectively; that is, prokaryotic phytoplankton are the origin of ~69% and ~67% of the PN at ALOHA and BATS. When expressed in C units, these contributions correspond to ~65% and 63%. These proportions are remarkably similar to those of living phytoplankton biomass assessments based on taxonomically sorted cell count data from flow cytometry, which indicate that approximately two-thirds of living phytoplankton biomass is prokaryotic (21, 22) (Fig. 4). High Δ_Chl-PN_ values were previously reported from an oligotrophic gyre station in the western North Pacific (−2.0 ± 2.2‰), consistent with the predominance of prokaryotic phytoplankton over the subtropical gyres (29).

In contrast to the subtropical gyre stations, Δ_Chl-PN_ values from the ETNP and Oregon coast are near the eukaryotic endmember (Δ_Chl-biomass_ = −5.5 ± 1.6‰) or lower, with mean values toward the lower end of the range, indicating that eukaryote-derived organic matter constitutes most of the suspended particle N pool in the surface waters of these regions (16–18). Cell-count biomass data from the same cruise along the Oregon coast show that eukaryotic phytoplankton constitute most of the living phytoplankton biomass (on average, 97.7 ± 1.5%). While the flow cytometry analysis largely excluded cells smaller than ~1 µm, including small cyanobacteria such as *Prochlorococcus* (see Methods), prokaryotic phytoplankton contribute minimally to the overall biomass in this high productive region (30), making the measurement limitation unimportant. The dominance of large cells in the phytoplankton community in the ETNP (23, 28), most of which are eukaryotic, also aligns with interpretation of the observed low Δ_Chl-PN_ values as reflecting a eukaryotic origin for most of the PN. Previously published Δ_Chl-PN_ values from other highly productive regions, such as the subarctic North Pacific (−5.4‰), the Southern Ocean (−4.2‰), and the Alboran Sea in the western Mediterranean (−5.3 ± 2.6‰), have been reported as negative and close to the eukaryotic endmember (16, 29, 31), consistent with typical phytoplankton populations in those regions as well (32–34) (*SI Appendix,* Table S1).

### Persistence of Prokaryotic Materials in the Extragyre North Atlantic.

Contrary to the similar estimates of prokaryotic-to-eukaryotic phytoplankton ratio from Δ_Chl-PN_ and cell counts in the subtropics and at the eastern North Pacific margin, a significant deviation of Δ_Chl-PN_ from cell-count biomass data is observed at the temperate-to-subpolar North Atlantic stations of EN538 and EN532, as well as at the subtropical-tropical eastern North Atlantic stations of SYES. At these North Atlantic stations, Δ_Chl-PN_ is higher (i.e., a lower amplitude negative value) than predicted if the total particle pool followed the fraction of eukaryotes in the living phytoplankton community observed by flow cytometry (f_euk,phyto_, Fig. 4*A*). Thus, in the North Atlantic, f_euk,PN_ is markedly lower than f_euk,phyto_ (Fig. 4*B*), even when considering the uncertainty range of the Δ_Chl-biomass_ isotopic endmembers (*SI Appendix*). That is, the prokaryotic phytoplankton account for a larger fraction of the suspended PN than of the living phytoplankton biomass.

The discrepancy is most pronounced in the EN538 samples, which were collected during the early phase of the spring bloom (May 2014). At all EN538 sampling stations, cell-count biomass data reveal the dominance of eukaryotes in living phytoplankton communities (f_euk,phyto_ >90%). However, Δ_Chl-PN_ exhibits a wide range, covering the entire spectrum of prokaryotic and eukaryotic phytoplankton endmembers (Fig. 4*A*). The f_euk,PN_ is on average 0.61 ± 0.33, much lower than f_euk,phyto_ (0.98 ± 0.02). As the cell-count biomass data exclude the large cells (of >14 µm in diameter), the total living algal community likely contains an even higher fraction of eukaryotes, suggesting an even greater difference between f_euk,PN_ and f_euk,phyto_.

Across the EN538 sampling stations, some Δ_Chl-PN_ values closely resemble those of pure cyanobacteria (Δ_Chl-biomass_ = 0.2 ± 2.5‰), even when >90% of the flow cytometry-sorted living cell biomass is eukaryotic. The difference between Δ_Chl-PN_ and the value that would correspond to the living phytoplankton population suggests that nonliving material dominated the suspended PN. This inference is supported by the observation that the flow cytometry-sorted phytoplankton cell biomass from the EN538 samples represents only a minor fraction (on average, 18.5 ± 12.1%) of the total particulate organic carbon (POC) (*SI Appendix,* Table S2). If heterotrophic bacteria assimilate recycled N with low δ^15^N and contribute their biomass to PN, they could lower the bulk PN δ^15^N and thereby increase Δ_Chl-PN_. However, the reliance of heterotrophic bacteria on recycled N is uncertain, particularly in environments where nitrate and/or PN is available, such as at most EN538 stations. Therefore, the N isotope data require that essentially all of the remaining POC was derived from prokaryotic phytoplankton (*SI Appendix*).

The combined sinks for PN in subtropical surface waters do not appear to involve significant net isotopic fractionation (*SI Appendix,* Fig. S3) (21, 35). Nevertheless, if such fractionation did apply, then it would shift measured Δ_Chl-PN_ to lower (more negative) values, implying that the true discrepancies between f_euk,PN_ and f_euk,phyto_ are even greater than indicated. That is, the noted discrepancies cannot be an artifact of trophic or diagenetic elevation in the δ^15^N of PN, and any occurrence of these processes would only strengthen our evidence for a bias toward prokaryotic phytoplankton in the dead particles of the surface ocean. Similarly, subsurface particles may enter the surface particle pool through upwelling, vertical mixing, or episodic eddies. The vertical changes in δ^15^N_PN_ and δ^15^N_Chl_ indicate that, while δ^15^N_PN_ generally increases with depth, subsurface δ^15^N_Chl_ remains similar to surface values, yielding more negative Δ_Chl-PN_ signatures (i.e., eukaryote-like; Fig. 2). Consequently, input from subsurface particles would also raise f_euk,PN_, further increasing the need for an explanation for the measured low f_euk,PN_ relative to f_euk,phyto_.

In contrast to the cell-count data from EN538, the chlorophyll a concentration for the small size fraction (<2 µm) exhibits a strong correlation with Δ_Chl-PN_ (*SI Appendix,* Fig. S4). Size-fractionated chlorophyll derives from both living and phytodetrital materials, as do the chlorins and bulk suspended particles measured for δ^15^N, explaining the better agreement. However, there is also some disagreement between chlorophyll size fractions and Δ_Chl-PN_, with the latter indicating a greater fraction of prokaryotic material. This discrepancy may be due to the exclusion of phaeopigments (chlorophyll degradation products) in the size-fractionated chlorophyll measurements, vs. their inclusion in the Δ_Chl-PN_ measurements. Thus, a bias for the retention of dead prokaryotic particles in the surface ocean could explain both the better correlation of Δ_Chl-PN_ with size-fractionated chlorophyll than with f_euk,phyto_ and the tendency of f_euk,PN_ to be somewhat greater than expected from size-fractionated chlorophyll a (*SI Appendix,* Fig. S4).

The EN538 samples were collected during the early stages of the spring bloom in the temperate-to-subpolar North Atlantic (36). The timing of this sampling raises the possibility that the prespring bloom ecosystem contained a higher proportion of prokaryotes and that this material persisted in the water column. The observed seasonal transition of phytoplankton community structure in the subpolar North Atlantic aligns with this possibility, shifting from prokaryote dominance in winter to eukaryote dominance in spring (37). Moreover, nitrate concentration is positively correlated with prokaryote-sourced PN, estimated by multiplying suspended PN concentration by (1 – f_euk,PN_) (*SI Appendix,* Fig. S5). This suggests that regions with less-developed blooms may have retained more prokaryote-sourced PN from before the bloom. In general, this raises the possibility that the bias toward the persistence of prokaryote-sourced materials in surface waters observed across our dataset can be explained solely by seasonal succession, i.e., the carryover of dead prokaryotic material from the prebloom season. However, given a turnover time of suspended particles in the water column of 1 to 2 wk (38), it is unlikely to explain the entire discrepancy, especially in cases where nearly all the nitrate had been consumed (*SI Appendix,* Fig. S6). Indeed, at the EN538 stations, the ratio of measured living to total PN shows a positive correlation with Δ_Chl-PN_ (*SI Appendix,* Fig. S7), which suggests that the prokaryote bias occurs even at stations that are not dominated by dead, prebloom organic matter. Moreover, the bias toward persistence of prokaryote-sourced materials in the surface is also apparent in the EN532 samples collected in summer, although the bias is smaller (Fig. 4*B*). Accounting for the prokaryotic contribution to PN in EN532 (~28%) solely as a result of prebloom PN persistence until summer would require long turnover times exceeding 4 to 5 mo, which is implausible given rates of net primary production (NPP); indeed, based on NPP and POC stocks at the EN532 stations in the subpolar North Atlantic, a turnover time of 7 to 10 d is estimated (39). This indicates that the overrepresentation of prokaryotic (as opposed to eukaryotic) phytoplankton in nonliving suspended PN is not solely a seasonal occurrence but rather a prevailing phenomenon in the North Atlantic.

Finally, the large difference between f_euk,phyto_ and f_euk,PN_ in the eastern North Atlantic samples of SYES implies that this phenomenon extends to other nongyre North Atlantic environments. Lateral advection from the subtropical gyre could transport prokaryote-rich particles into temperate waters. However, the Δ_Chl-PN_ values in the temperate North Atlantic are even closer to the cyanobacterial Δ_Chl-PN_ endmember than are those in the subtropical gyre, ruling out transport alone as the cause of the low Δ_Chl-PN_ in the eastern North Atlantic.

### Different Fates of Prokaryotes and Eukaryotes in the Upper Ocean.

The bias toward a prokaryotic origin of suspended particles in North Atlantic surface waters can be explained by the preferential removal of eukaryotes from the upper ocean as sinking particles, presumably mostly as nonliving eukaryotic biomass (12). A comparison of PN δ^15^N values estimated from nitrate consumption (δ^15^N_PN,exp_, assuming Rayleigh-type isotope fractionation during nitrate assimilation) with observed δ^15^N_PN_ provides independent support for this interpretation. In the temperate North Atlantic stations of EN538, δ^15^N_PN,exp_ is generally higher than the observed δ^15^N_PN_, indicating preferential removal of high-δ^15^N PN (*SI Appendix,* Fig. S8, and Fig. S9). Previous studies comparing sinking and surface-suspended PN have found sinking PN to exhibit higher δ^15^N values (40), interpreting this difference as reflecting a contribution of higher trophic-level processes to sinking particle formation, such as zooplankton fecal pellet production. However, this difference may also be explained by the preferential removal of eukaryotic biomass, which tends to have higher δ^15^N due to its reliance on nitrate rather than recycled N (Fig. 3) (21). Inspecting the data in greater detail, Δ_Chl-PN_ is positively correlated with δ^15^N_PN,exp_ – δ^15^N_PN_, a relationship that is not well explained by autocorrelation from δ^15^N_PN_ variation (*SI Appendix,* Fig. S9). This correlation provides additional support for the selective export of eukaryotic phytoplankton-derived material, which is low in Δ_Chl-biomass_ and high in δ^15^N. In summary, the isotopic relationships of nitrate supply, bulk suspended PN, sinking PN, and the chlorins in suspended PN are consistent with the paradigm of the selective removal of eukaryotic phytoplankton-derived organic matter from the euphotic zone in sinking particles (21).

The hypothesis that eukaryotic biomass dominates the sinking particle flux originally arose in the context of particle size, affecting both particles’ vulnerability to be grazed and exported by zooplankton such as copepods (41) and their tendency to sink after death (10). The selective feeding of zooplankton on eukaryotic taxa (42) should preferentially incorporate eukaryotic phytoplankton biomass into rapidly sinking fecal pellets. However, zooplankton feeding on live cells cannot explain the difference between f_euk,PN_ and f_euk,phyto_, as this feeding should not directly affect live-to-dead ratio in the suspended PN pool. Instead, the data point to a greater tendency for eukaryotic debris to sink directly. Mechanistically, aggregation of large phytoplankton has been suggested to play a significant role in driving sinking export, especially in the North Atlantic (43). Due to the larger size of their cells, eukaryotes require less aggregation to reach particle sizes that sink rapidly into the ocean interior (10). Eukaryotic pigments dominate subsurface particle aggregates in the Sargasso Sea (12), supporting our interpretation.

**Fig. 5.**
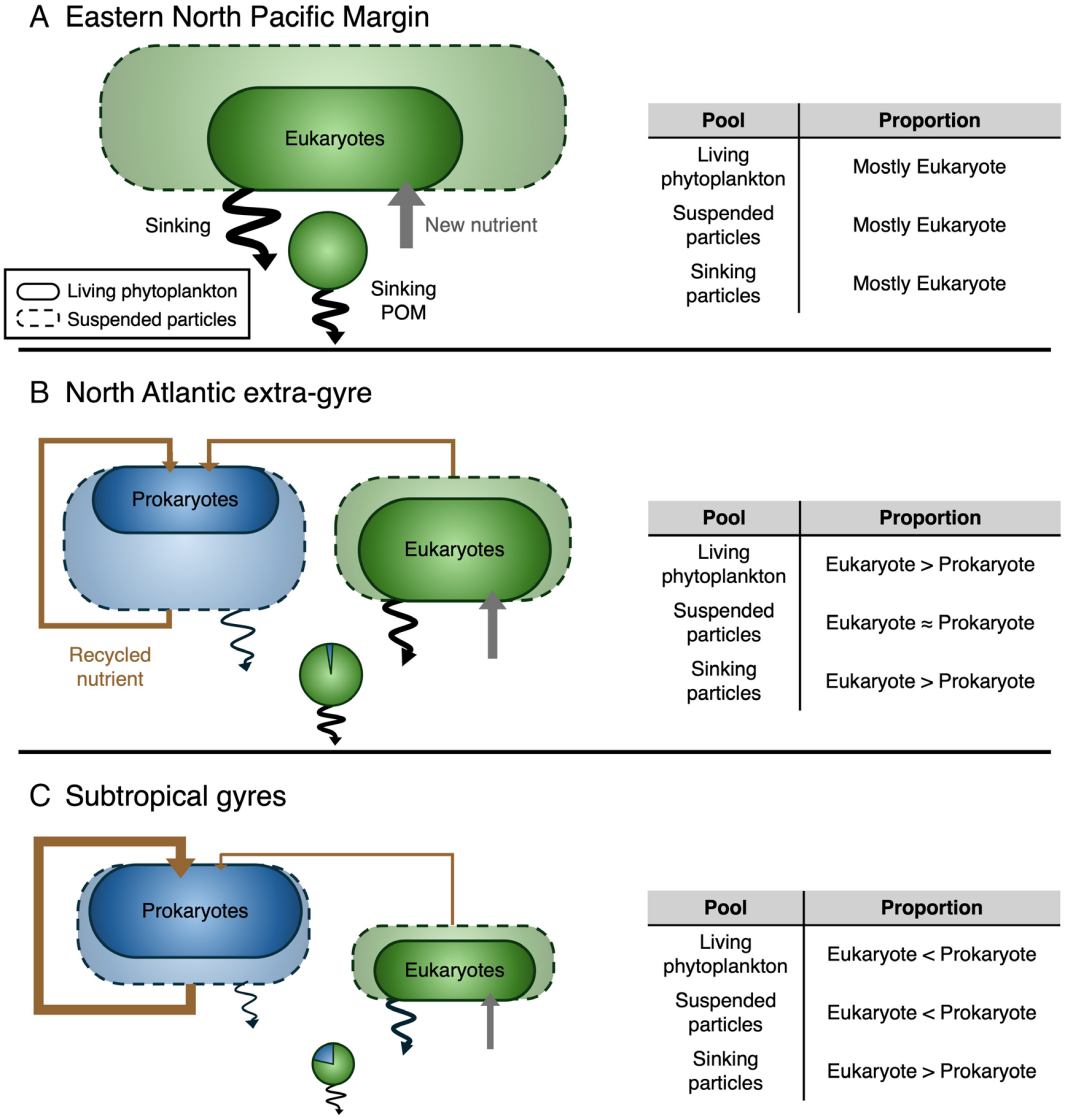
The eukaryote-to-prokaryote fraction in living phytoplankton, suspended particles, and sinking particles in different regions (*A*: eastern North Pacific margin, *B*: North Atlantic extragyre, *C*: subtropical gyres). The solid and dashed circles indicate living phytoplankton and suspended particles, respectively. Relative contribution of eukaryotic phytoplankton-originating vs. prokaryotic phytoplankton-originating organic materials to sinking particulate organic matter (POM) is denoted with the pie chart (green: eukaryote, blue: prokaryote). The size of the pie chart in each panel qualitatively represents the magnitude of export production. Gray arrows represent the input of new nutrients to the euphotic zone, while brown arrows indicate the flow of recycled nutrients. The wavy black arrow denotes the flux of sinking POM. The thickness of each arrow corresponds to the magnitude of the respective flux. Relative to prokaryotes, eukaryotes acquire a smaller fraction of their N from recycled pools (21); for simplicity, eukaryotes’ assimilation of recycled N is completely omitted here.

The contrast between the very strong bias toward prokaryote-derived organic matter in the North Atlantic extragyre and the lack of this bias in the subtropical gyres raises the prospect of an additional mechanism for the prokaryote bias in the former: slower recycling of prokaryote- relative to eukaryote-derived organic matter. Prokaryotic phytoplankton biomass has been proposed to include compounds that turn over slowly, such as peptidoglycan (44). Peptidoglycan is a constituent of bacterial cell walls and has been identified as a major contributor to marine dissolved organic N (45, 46), and this may extend to PN (47). The implied lack of accumulation of this recalcitrant prokaryotic debris in the subtropical gyres may be due to heightened demand for bioavailable N in the lowest nutrient settings, leading to more aggressive organic N degradation by heterotrophic bacteria in the subtropical gyres (Fig. 5*C*). However, as described above, the correlation between Δ_Chl-PN_ and the proportion of chlorophyll a in small particles (<2 µm) at EN538 (*SI Appendix,* Fig. S4) argues that there is a prokaryotic bias in both bulk N and chlorophyll a in surface waters. Considering that chlorophyll is a relatively labile component, the chemical recalcitrance of specific prokaryotic biochemicals cannot fully explain the observed prokaryotic bias in suspended particles. Preferential sinking of eukaryotic material is required and likely the dominant process driving the discrepancy between f_euk,PN_ and f_euk,phyto_.

We interpret the observed geographic variations in the “prokaryotic bias” as follows (Fig. 5). In highly productive environments such as along the eastern North Pacific margin, eukaryotic phytoplankton are essentially the only phytoplankton source of particles and so necessarily dominate the suspended particle pool, preventing an observable prokaryotic bias (Fig. 5*A*). This interpretation applies equally well to individual stations from other regions, such as a shallow coastal station of EN538 near the Grand Banks, where both f_euk,phyto_ and f_euk,PN_ are near 1 (*SI Appendix,* Fig. S6). In the intermediate productivity stations of the nonsubtropical-gyre North Atlantic, where there is a substantial prokaryotic component to primary production (37), preferential export of eukaryotic material lowers f_euk,PN_ (Fig. 5*B*). In the subtropical gyres, the low primary productivity and scarcity of organic matter and N drive the most aggressive, efficient degradation of prokaryotic biomass by the microbial loop, counteracting the effect of preferential eukaryotic PN export (Fig. 5*C*). The observed high phytoplankton biomass N-to-PN (and biomass C-to-POC) ratio in the Sargasso Sea, compared with other regions (*SI Appendix,* Table S2), provides additional support for such efficient recycling of nonliving suspended POM in this oligotrophic environment and would also work to minimize the discrepancy between f_euk,phyto_ and f_euk,PN_. In this interpretation, the regions and stations of intermediate productivity, due to their combination of a prokaryotic phytoplankton population and a significant concentration of dead POM, are able to show the “prokaryotic bias”, providing a unique window into the different fates of prokaryotic and eukaryotic phytoplankton biomass in the surface ocean. Regional variations aside, the accumulated observations support the paradigm that eukaryotic phytoplankton play an outsized role in the biological pump (21) while prokaryotic phytoplankton preferentially fuel the upper ocean’s microbial loop and the organisms that this loop supports (48, 49).

Prokaryotic and eukaryotic phytoplankton have distinct environmental sensitivities (50, 51). Accordingly, upper ocean stratification under global warming is projected to increase the relative importance of prokaryotic phytoplankton (52). Considering the different fates of prokaryotic and eukaryotic phytoplankton biomass implied by our data, this change should work to increase the retention of organic matter in surface waters, potentially incorporating more prokaryote-derived material into longer-term organic matter cycling, including that of dissolved organic matter (45, 46, 53). At the same time, it would reduce the efficiency of organic matter export, with significant implications for both surface ocean ecosystems and the ocean interior (54). Moreover, a rise in the prokaryote-to-eukaryote ratio may work to reduce the predicted increase in the completeness of nutrient consumption in temperate to subpolar ocean surface waters (55). If so, the eukaryote-to-prokaryote shift would act as a negative (stabilizing) feedback that mutes changes in the nutrient distributions of the upper ocean. Looking backward in time, our findings support the view that deep ocean sediments better represent eukaryotic phytoplankton than prokaryotic phytoplankton (19, 56). Finally, our findings inform the effort to reconstruct the evolution of the ecology and biogeochemistry of the ocean as it transitioned from prokaryotic dominance to a mixed and variable importance of prokaryotic and eukaryotic phytoplankton and finally to the modern state that includes invertebrate zooplankton grazing (57).

## Materials and Methods

### Sample Collection.

Samples were collected from 9 different sites and/or cruises: a Station ALOHA occupation in June 2019, three stations in the eastern tropical North Pacific (ETNP) in March 2018, eight stations of the PUPCYCLE (Phytoplankton response to the UPwelling conveyor belt CYCLE) II cruise over the Oregon Coast occupation in May 2023, a BATS (Bermuda Atlantic Time-series Study) station occupation in June 2019, stations from 9 cruises of the SYES (S/Y *Eugen Seibold*) in the eastern North Atlantic (1 station in August 2019, 6 stations in July–November 2020, and 2 stations in April–May 2021; SYES 2019-2021 stations), 2 stations in the subpolar North Atlantic in September 2013 (during the EN532 cruise), and 13 stations during the EN538 cruise in the subtropical and subpolar North Atlantic in May 2014 (Fig. 1*A*). Specifically, ALOHA, BATS, PUPCYCLE II, and ETNP samples were collected from ~5 m via underway intake; EN532 and EN538 samples were collected from the upper 50 m; and SYES samples were generally collected from within the upper 50 m, with three exceptions extending to 103 m. At ALOHA and SYES, additional samples from the subphotic zone were collected to analyze the vertical distributions of δ^15^N_PN_ and δ^15^N_Chl_. Detailed information is provided in *SI Appendix,* Table S3. Across all sampling regions, *Trichodesmium*, other colonial phytoplankton, and macroalgae were not observed.

Particles were collected using precombusted (450°C, >5 h) glass fiber filters, which were frozen immediately and stored in an −80°C freezer. In the case of the 2 stations in the subpolar North Atlantic and the 13 stations of the EN538 cruise, about 10 L of water was filtered through a GF-75 filter (47 mm diameter, 0.3 µm pore size, Advantec). For the SYES 2019-2021 stations, around 100 L of water was filtered through a GF-75 filter (142 mm diameter, 0.3 µm pore size, Advantec) with an in-situ pump (McLane, WTS-LV) and stored at −20°C immediately after recovery. For sampling at ETNP, ALOHA, and BATS stations, a 51 µm acid-washed nylon mesh screen prefilter (Nitex brand) was used, in-line with a GF-75 filter (142 mm diameter, 0.3 µm pore size, Advantec) connected to the shipboard underway pumping system. In the PUPCYCLE II cruise stations, 10 to 20 L of water from the shipboard underway pumping system was filtered through a GF-75 filter (142 mm diameter, 0.3 µm pore size, Advantec). At Station ALOHA and the BATS station, where the surface chlorophyll concentration is relatively low, approximately 300 L of water was filtered to obtain sufficient chlorophyll for analysis. Furthermore, a 1.2 µm glass fiber filter (142 mm diameter, Whatman GF/C) was included between the 51 µm mesh screen prefilter and GF-75 filter at these stations. These samples were collected with the original intent to measure nitrogen isotopes in the different size fractions. However, most of the total suspended PN (>85%) was collected on the 1.2 µm filter, possibly due to particle loading reducing the effective filter pore size. Thus, we report the bulk and chlorin δ^15^N values of suspended particles collected on the 1.2 µm filters from Station ALOHA and BATS. Our δ^15^N analyses show that the δ^15^N of bulk PN and chlorin on the 1.2 µm filters were not different from those of the total suspended PN collected via nonsize fractionated filtration (i.e., direct filtration onto GF-75 filters).

For subsurface sampling, at Station ALOHA, particles were collected in two ways. First, McLane pumps filtered ~650 L through a 51 µm nylon prefilter followed by 142 mm GF/F filters, and metal punches of ~13 to 17 L per punch were used for subsampling. Second, additional samples from the deep chlorophyll maximum (~120 m) were collected with Niskin bottles and filtered (~24 L) through a 51 µm prefilter followed by a 1.2 µm GF/C filter. SYES subsurface samples were collected using the same approach as surface samples (~100 L filtered through 142 mm GF/F filters, 0.7 µm pore size, with in-situ McLane pumps). Detailed information on the samples and filtration is provided in *SI Appendix,* Table S4.

### Bulk Particle δ^15^N Analysis.

The filter samples were freeze-dried at −55°C overnight (>24 h). For bulk PN δ^15^N analysis, the filters were subsampled with a 5 mm diameter punch and placed into precombusted (500°C, >5 h) 4 mL borosilicate glass vials. Potassium persulfate (K_2_S_2_O_8_) was purified through three recrystallization and dissolution cycles. Persulfate oxidation reagent (POR) solution was prepared by dissolving 1 g of the purified potassium persulfate and 1 g of sodium hydroxide (NaOH) in 100 mL of distilled, deionized water (Milli-Q) (58). The subsampled filter was treated with 50 μL of 4 N hydrochloric acid (HCl (aq)) to dissolve any particulate carbonate. POR was then added to each vial, and the tightly capped vials were autoclaved at 120°C under 12 psi for 55 min. The concentration of organic nitrogen that was oxidized to NO_3_^−^ was measured by conversion to NO followed by chemiluminescent detection (59) (Teledyne NO/NOx Analyzer 200E), and its N isotope ratio was analyzed with the denitrifier method (60).

### Chlorin Extraction and δ^15^N Analysis.

Chlorin from filter samples were extracted according to refs. 18 and 61, with a simple but important modification of the POR recipe as indicated below. Briefly, filter samples were soaked in a 2:1 (v/v) mixture of dichloromethane and methanol (DCM/MeOH) with sonication for 20 min in an immersion bath. The extracts were added to precombusted silica gel columns with sodium sulfate (Na_2_SO_4_) and eluted with DCM/MeOH (2:1 v/v). The extracts were further purified with an HPLC (Agilent 1200 series) equipped with a multiwavelength UV/Vis detector. Samples were injected into a ZORBAX SIL column (4.6 × 250 mm) and eluted at 1 mL min^−1^ using the polarity gradient (61).

Chlorins were identified using absorbance spectra and comparison to authentic standards of chlorophyll a (Sigma Aldrich), bacteriochlorophyll a (Sigma Aldrich), pheophorbide a ([PHA-592], Frontier Science), and two porphyrin compounds (Octaethylporphyrin (O534), Vanadyl Octaethylporphine (VO-OEP), both from Frontier Science). The purified chlorin fraction was collected using time-based fraction collection from 8 to 14 min, then fully dried under high-purity N_2_ gas (>99.999%) and reconstituted in DCM in a 4 mL borosilicate glass vial. While the PHA-592 exhibited its primary peak at 17 min during the HPLC run, our filter samples displayed main peaks at around 10 min. Consequently, we conducted an analysis on the collected fractions spanning the 8 min to 14 min range. The vials were placed under UV light in a biosafety cabinet (Labconco Class II type A2) for 6 h with their Teflon-lined caps tightly closed. This step was included by ref. 61 to maximize the oxidation of porphyrin, a degradation product of chlorophyll. The chlorin extract was again dried with high-purity N_2_ and then oxidized to NO_3_^−^ by reaction with 0.05 M K_2_S_2_O_8_ dissolved in a 0.5 M NaOH solution. To test the oxidation yield, two porphyrin standards (O534 and VO-OEP) were also chemically oxidized, and the resultant concentrations were compared with the expected yields. The concentration of the NO_3_^−^ product was measured by chemiluminescence (as above) and its δ^15^N was analyzed with the denitrifier method (60).

Ref. 61 reported a low yield for the chemical oxidation of porphyrin by POR without exposure to UV. However, in our methods testing, we observed near 100% oxidation yield of the same porphyrin standard (VO-OEP) without UV treatment when we used a different POR recipe to that of ref. 61 (*SI Appendix,* Fig. S10). The new recipe yielded a more basic condition for the POR, which we suspect explains the more complete NO_3_^−^ yield from porphyrin. Despite this finding, we still exposed all our samples to UV for 6 h, following the protocol of Ref. 61,58, to err on the side of caution regarding complete oxidation. The δ^15^N measurements of the chlorin standards with persulfate oxidation followed by the denitrifier method were compared with δ^15^N measurements of larger quantities of these standards made using an Elemental Analyzer-Isotope Ratio Mass Spectrometer (EA-IRMS; Elementar Isoprime visION). The δ^15^N measurements from the two methods agree within 0.4‰ for the three chlorins (*SI Appendix,* Table S5).

The average procedural blank was determined to be 1.4 nmol N in a 1 mL sample, which comprises less than ~8% of the total N in the measurement. The blank could have originated from various sources, including the solvent used for HPLC and UV oxidation (n_sol_), the POR solution (n_por_), and/or the denitrifier and isotopic analysis protocols (n_den_) (61). While the solvents used for extraction and flash chromatography might contain N-bearing contaminants, the HPLC step should remove these. As a result, only the solvent blank from the HPLC and UV oxidation steps (n_sol_) was considered. The average n_por_ and n_den_ were found to be 0.3 nmol N and 0.1 nmol N, respectively, leaving the remaining 1.0 nmol N blank associated with the solvent (n_sol_). The quantification of n_sol_ with respect to the solvent volume added during the HPLC and UV oxidation steps resulted in a value of 0.13 nmol N per 1 mL of solvent, which aligns with the previously reported blank value for this method (61). The δ^15^N measurements for chlorin standards without the UV treatment exhibited reproducibility with a 1SD below 0.3‰. With UV treatment, the 1SD was slightly higher (<0.5‰).

### Flow Cytometry-cell Count Biomass Analysis.

Cell abundance of phytoplankton for the Oregon coast samples was newly determined with flow cytometry in this study. Seawater for flow cytometry analysis was collected from CTD Niskin bottles and filtered through 52 µm mesh into 15 mL falcon tubes. The filtered samples were transferred in duplicates to a 96-well plate, and taxonomic populations were examined using a Guava easyCyte HT flow cytometer (Cytek Biosciences) at a flow rate of 0.59 µL s^−1^. Samples were run for 210 s (approx. 123.6 µL analyzed) or until a termination count of 20,000 events. The populations of *Synechococcus*, pico- and nano-eukaryotes were detected based on forward scatter (FSC) and red, yellow, and green fluorescence (62). Cryptophyte populations were identified based on high yellow and high red fluorescence. Cells smaller than ~1 µm were excluded due to insufficient instrument sensitivity, and cells larger than ~20 µm were excluded from the analysis. Carbon biomass estimates were derived from cell abundance using published conversion factors [100 to 250 fg C cell^−1^ for *Synechococcus*; eukaryotic phytoplankton: pg C = 0.433 × (biovolume)^0.863^] (63, 64). Due to the lack of generalized published cryptophyte carbon biomass values, cryptophyte carbon biomass was approximated in low, median, and high ranges (65), and the median carbon biomass values were used for comparison.

Cell abundance for the SYES samples was determined using a CytoSense online continuous flow cytometer using a 488 nm laser with, among others, 685 nm (red) and 631 nm (orange) band-pass filters (CytoBuoy). Samples at depth were collected from a rosette sampler deployed at the same station as ISP deployments and passed immediately to the flow cytometer for measurements of 5 to 10 min at 5 µL s^−1^ triggered on red fluorescence at 5 mV at high gain (20, 66). The SYES sample at 29°N lacks cell-count biomass data; for this sample, we use flow cytometry data from the same depth at a nearby station that was collected in the season month late October-early November two years later. Due to potential instrument fouling that caused misalignment in fluorescence and scatter measurements, the cell-count biomass data for the SYES sample at 40°N should be interpreted with caution. Nevertheless, the abundance profile remains generally consistent with the corresponding CTD data.

Detailed description for cell abundance analysis of EN532 (25) and EN538 samples are available in the BCO-DMO (https://www.bco-dmo.org/dataset/564097).

## Supplementary Material

Appendix 01 (PDF)

## Data Availability

All study data are included in the article and/or *SI Appendix*.
