# Supplementary material for: Prokaryotic bias in surface ocean particles
Source: Proc Natl Acad Sci U S A. 2026 Apr 1;123(14):e2500624123. doi: 10.1073/pnas.2500624123 (PMC13056086; doi:10.1073/pnas.2500624123)
Supplement: Supplementary file 1 — Appendix 01 (PDF) [file pnas.2500624123.sapp.pdf]

PNAS

Supporting Information for
Prokaryotic bias in surface ocean particles

Yeongjun Ryu[1,2]*, Ashley E. Maloney[1,3], Victoria H. Luu[1], Lingkun Guo[1], Sergey Oleynik[1], Sarah E. Fawcett[4,5], Meytal B. Higgins[6], Nicolas Van Oostende[1], Bess B. Ward[1], Claire C. Z. Cook[7], Natalie R. Cohen[7], Erica Ewton[8], Susanne Menden-Deuer[8], Julie Granger[9], Adrian Marchetti[10], Hedy M. Aardema[2,11], Hans A. Slagter[2], Ralf Schiebel[2], Alfredo Martínez-García[2], Gerald H. Haug[2,11], and Daniel M. Sigman[1]

[1]Department of Geosciences, Princeton University; Princeton, New Jersey, 08544, USA
[2]Climate Geochemistry Department, Max-Planck Institute for Chemistry; Mainz, 55128, Germany
[3]Department of Geological Sciences, University of Colorado; Boulder, Colorado, 80309, USA
[4]Department of Oceanography, University of Cape Town; Rondebosch, 7701, South Africa
[5]Marine and Antarctic Research Centre for Innovation and Sustainability (MARIS), University of Cape Town; Rondebosch, 7701, South Africa
[6]ExxonMobil Technology and Engineering Company; Annandale, New Jersey, 08801, USA
[7]Skidaway Institute of Oceanography, University of Georgia; Savannah, Georgia, 31411, USA
[8]Graduate School of Oceanography, University of Rhode Island; Narragansett, Rhode Island, 02882, USA
[9]Department of Marine Sciences, University of Connecticut; Groton, Connecticut, 06340, USA
[10]Department of Earth, Marine and Environmental Sciences; University of North Carolina, Chapel Hill, North Carolina, 27514, USA
[11]Department of Earth and Planetary Sciences, ETH Zürich; Zürich, 8092, Switzerland

Email: yeongjun@princeton.edu

This PDF file includes:

Supporting text
Figures S1 to S10
Tables S1 to S5

Supporting Information Text

$\delta^{15}N$ of bulk suspended PN and chlorins reflecting phytoplankton origins

Chlorins originate dominantly from the chlorophyll a of phytoplankton, from which alteration occurs. Such alteration, if it has any isotopic effect, would simply redistribute N isotopes within the chlorin pool, leaving the $\delta^{15}N$ of the total chlorin pool unchanged. Moreover, both chlorin alteration and the loss of chlorins from the analytical window should occur without significant isotopic fractionation (1,2), and existing data support this expectation (3).

Phytoplankton photosynthetic growth is the ultimate origin of all suspended PN. However, heterotrophic organisms (especially bacteria) are in the PN pool (4), while non-living particles dominate the PN pool (5). These forms of PN could be distinct from the original PN produced by phytoplankton, for example, due to low-$\delta^{15}N$ N loss in metabolic and diagenetic processes. However, the available $\delta^{15}N$ measurements of flow cytometrically sorted particles from the subtropical gyre systems indicate that heterotrophic bacteria have similar $\delta^{15}N$ to the prokaryotic phytoplankton (6) (Fig. S3). Protistan grazers, the other major contributor to heterotrophic biomass in surface waters, have also been observed to have a $\delta^{15}N$ similar to (not substantially higher than) their organic matter source (7). The $\delta^{15}N$ of non-living organic matter can rise due to isotopic fractionation by degradation (8). However, bulk suspended PN in the euphotic zone is observed to have a $\delta^{15}N$ similar to (and not higher than) the total living plankton measured by flow cytometry (6) (Fig. S3). This suggests that suspended PN is lost mostly by non-fractionating processes, such as by feeding or by disaggregation or initial decomposition by the break-off of large macromolecules (9), which leads to little net isotopic fractionation of the total N pool in the substrate and product (2). In summary, the data indicate that the bulk suspended PN $\delta^{15}N$ in the euphotic zone reflects the $\delta^{15}N$ of its autotrophic origins.

Derivation of the $\Delta_{Chl\text{-}PN}$ mass balance equation

We can consider a model similar to that shown in Fig. 3, based on previous studies in the Sargasso Sea (6). In this model, the $\delta^{15}N$ difference between eukaryotic and prokaryotic phytoplankton biomass is roughly comparable to the difference in their isotopic fractionation associated with chlorophyll biosynthesis. This $\delta^{15}N$ difference between eukaryotic and prokaryotic phytoplankton biomass is consistently observed even in the subpolar North Atlantic (10), where nutrient conditions are very different from the Sargasso Sea. This convergence of $\delta^{15}N_{Chl}$ between eukaryotes and prokaryotes simplifies the mathematical formulation of our model and makes it insensitive to variations in Chl:C ratios. The mass balance equations for PN and chlorin are written as follows:

$$\delta^{15}N_{PN} = f_{euk,PN} * \delta^{15}N_{euk,PN} + f_{pro,PN} * \delta^{15}N_{pro,PN}$$
$$\delta^{15}N_{Chl} = f_{euk,Chl} * \delta^{15}N_{euk,Chl} + f_{pro,Chl} * \delta^{15}N_{pro,Chl}$$

These equations express the $\delta^{15}N$ values of bulk PN and chlorins as contributions from eukaryotic and prokaryotic phytoplankton sources. Here, the terms $f_{euk,PN}$ and $f_{euk,Chl}$ represent the fractional contributions of eukaryotes to PN and chlorins, respectively, which may differ.

From these definitions, we can describe $\Delta_{Chl\text{-}PN}$, the difference between the $\delta^{15}N$ of chlorins and that of bulk PN, as follows:

$$\begin{aligned}\Delta_{Chl\text{-}PN} &= \delta^{15}N_{Chl} - \delta^{15}N_{PN}\\ &= (f_{euk,Chl}*\delta^{15}N_{euk,Chl} + f_{pro,Chl}*\delta^{15}N_{pro,Chl}) - (f_{euk,PN}*\delta^{15}N_{euk,PN} + f_{pro,PN}*\delta^{15}N_{pro,PN})\\ &= (f_{euk,Chl}*\delta^{15}N_{euk,Chl} + (1 - f_{euk,Chl})*\delta^{15}N_{pro,Chl}) - (f_{euk,PN}*\delta^{15}N_{euk,PN} + (1 - f_{euk,PN})*\delta^{15}N_{pro,PN})\end{aligned}$$

If we assume that $\delta^{15}N_{Chl}$ values are similar between eukaryotes and prokaryotes (i.e., $\delta^{15}N_{euk,Chl} = \delta^{15}N_{pro,Chl}$), as suggested by the convergence observed in our data:

$$\begin{aligned}&= \delta^{15}N_{Chl} - (f_{euk,PN}*\delta^{15}N_{euk,PN} + (1 - f_{euk,PN})*\delta^{15}N_{pro,PN})\\ &= f_{euk,PN} * (\delta^{15}N_{Chl} - \delta^{15}N_{euk,PN}) + (1 - f_{euk,PN})*(\delta^{15}N_{Chl} - \delta^{15}N_{pro,PN})\\ &= f_{euk,PN}*\Delta_{Chl\text{-}PN,euk} + (1 - f_{euk,PN})* \Delta_{Chl\text{-}PN,pro}\end{aligned}$$

Thus, the final expression is independent of the Chl:C ratio and can be written purely as a function of the group-specific $\Delta_{Chl\text{-}PN}$ values for eukaryotic and prokaryotic phytoplankton (Equation 1 in the main text). Therefore, the similarity in $\delta^{15}N_{Chl}$ between the two groups provides mathematical justification for using $\Delta_{Chl\text{-}PN}$ as a robust indicator of the relative nitrogen contributions of eukaryotes and prokaryotes to bulk PN.

Sensitivity of $f_{euk,PN}$ calculations to $\Delta_{Chl\text{–}biomass}$ endmember variability

Estimates of $f_{euk,PN}$ depend on the offsets between chlorophyll and biomass $\delta^{15}N$. In this study, we use mean $\Delta_{Chl\text{-}biomass}$ values from culture experiments for the eukaryotic and prokaryotic endmembers ($\Delta_{Chl\text{-}biomass}$ = -5.5 ± 1.6‰ for eukaryotes and 0.2 ± 2.5‰ for prokaryotes) in all calculations presented in the main text. Considering the uncertainty ranges of these endmembers, the absolute $f_{euk,PN}$ values may be affected. For instance, if the eukaryotic endmember were at its upper bound (−3.9‰) and the prokaryotic endmember at its lower bound (−2.3‰), the resulting difference between endmembers would decrease to 1.6‰. In this case, equation (3) in the main text becomes:

$$f_{euk,PN} = \frac{\Delta_{Chl-PN} + 2.3‰}{-1.6‰}$$

Considering this uncertainty, only $\Delta_{Chl\text{-}PN}$ values above a threshold (−3.9‰) would yield $f_{euk,PN} < 1$. Even accounting for the $\Delta_{Chl\text{-}biomass}$ isotopic uncertainty, many North Atlantic samples (e.g., SYES, EN538) have $\Delta_{Chl\text{-}PN\ values}$ below −3.9‰, indicating that $f_{euk,PN}$ remains substantially lower than $f_{euk,phyto}$ and supporting our main interpretation.

Comparison between $f_{euk,PN}$ and $f_{euk,phyto}$ from previous studies

Previously published $\Delta_{Chl\text{-}PN}$ values are consistent with phytoplankton population estimates. For high productive regions such as the subarctic North Pacific, the Southern Ocean, and the Alboran Sea in the western Mediterranean, have been reported as negative and close to the eukaryotic endmember (11-13), whereas the western North Pacific subtropical gyre shows higher $\Delta_{Chl\text{-}PN}$, consistent with typical phytoplankton populations in those regions (Table S1). $\Delta_{Chl\text{-}PN}$ data from the Arabian Sea, equatorial Eastern Pacific, and eastern Mediterranean (11,12) also suggest a dominant contribution of eukaryotic phytoplankton to the PN pool. Given the high temporal variability and dynamism of these regions and the lack of coincident observations of phytoplankton populations, it is unclear whether the co-occurring phytoplankton pool was also dominated by eukaryotes.

Comparison between flow cytometry sorted cell biomass C and suspended POC

To compare our measurements of suspended PN concentration with the flow cytometry-sorted cell biomass C, we must convert suspended PN concentration into suspended POC. Given the range of suspended particle C:N ratios in the ocean (5.5–9), suspended POC concentration was estimated as [POC] = [PN] × C:N (14).

Across the sampling sites, the fraction of flow cytometry sorted phytoplankton biomass C ($[POC]_{phyto}$) to the suspended POC ([POC]) varies widely, but in most cases, $[POC]_{phyto}/[POC]$ accounts for a minor fraction (Table S2). Samples from the Oregon coast show the lowest $[POC]_{phyto}/[POC]$, consistent with the large contribution of non-living particles in the coastal regions, which can exceed living phytoplankton fraction by 10-fold (5). In contrast, the flow cytometry sorted phytoplankton biomass N from the Sargasso Sea recovers about 50% of total suspended PN (6).

Using both the $f_{euk,phyto}$ and $[POC]_{phyto}$ from flow cytometry sorted cell data and $f_{euk,PN}$ and $[POC]_{total}$ from N isotope measurements, we calculate $f_{euk,res}$, the eukaryotic fraction of residual POC (e.g., not recovered with flow cytometry), which may include heterotrophic biomass and non-living material, in the suspended particles with mass balance equations described below.

$$[POC]_{res} = [POC] - [POC]_{phyto} \quad \text{(Eqn. S1)}$$

$$f_{euk,res}*[POC]_{res} = f_{euk,PN}*[POC] - f_{euk,phyto}*[POC]_{phyto} \quad \text{(Eqn. S2)}$$

$$f_{euk,res} = (f_{euk,PN}*[POC] - f_{euk,phyto}*[POC]_{phyto})/[POC]_{res} \quad \text{(Eqn. S3)}$$

If the $[POC]_{res}$ accounts for the dominant portion of [POC] (e.g., $[POC]_{res} \approx [POC]$), $f_{euk,rem}$ will be close to $f_{euk,PN}$. For instance, while all EN538 samples exhibit $f_{euk,phyto}$ values greater than 0.9, a few stations have $f_{euk,PN}$ close to zero (see Fig. 4). Given that the uncertainty of $f_{euk,PN}$ derived from $\Delta_{Chl\text{-}biomass}$ endmembers is approximately 30% and with an average of $[POC]_{phyto}/[POC]$ is 0.2 (thus, $[POC]_{res}/[POC] = 0.8$), we can calculate the $f_{euk,res}$ with the above equations (Eqn. S1-S3).

$$f_{euk,rem} = ((0 \pm 0.3)*[POC] - 0.9*[POC]*0.2)/([POC]*0.8)$$
$$f_{euk,res} = 0\text{-}0.15$$

This result indicates almost all (>85%) of the POC that excludes the living phytoplankton in EN538 samples is consistent with a prokaryotic phytoplankton origin. A fraction of this remaining POC could consist of heterotrophic biomass assimilating recycled N, which cannot be fully ruled out. However, the extent to which heterotrophs rely on recycled N may vary across locations, requiring further investigation. Overall, to explain the patterns observed across the broad North Atlantic transect, a prokaryote-sourced POC is the most likely explanation.

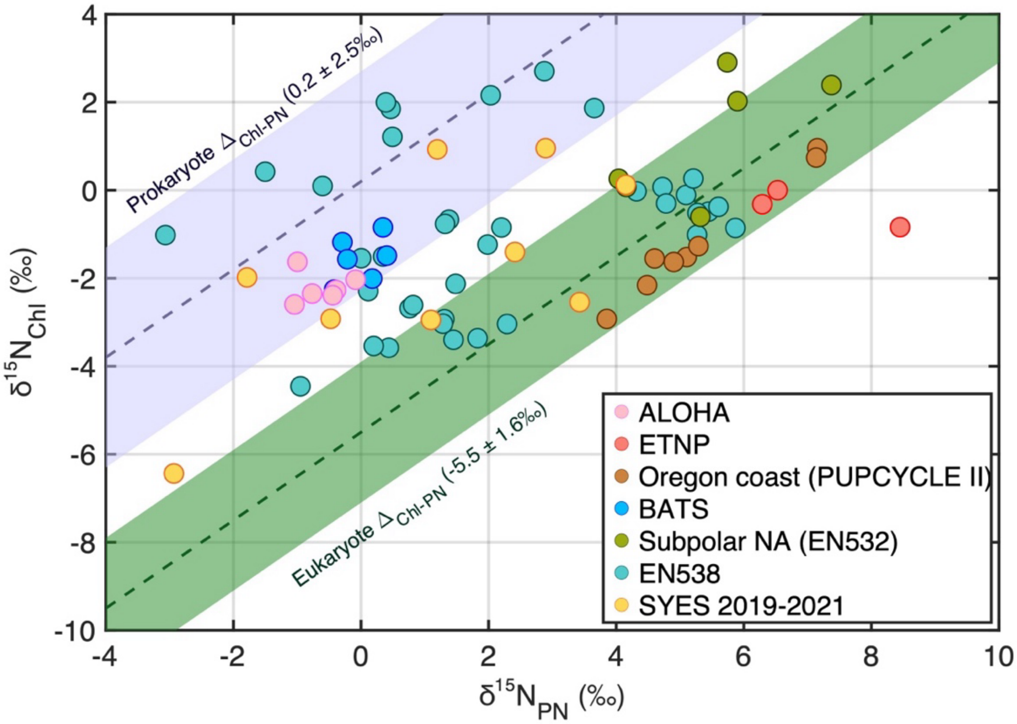

Fig. S1. δ^{15}N of bulk particulate nitrogen ($\delta^{15}N_{PN}$) and chlorin ($\delta^{15}N_{Chl}$) in the surface ocean (0-50 m). Colored circles are the data obtained from this study (pink: Station ALOHA, red: ETNP, brown: Oregon coast stations from PUPCYCLE II, blue: BATS Station, yellow: eastern North Atlantic stations from SYES 2019-2021, green: subpolar North Atlantic stations from EN532, cyan: North Atlantic stations from EN538). Two Δ_{Chl-PN} endmembers are represented by dashed lines with colored shading (green for eukaryotes, dark blue for prokaryotes).

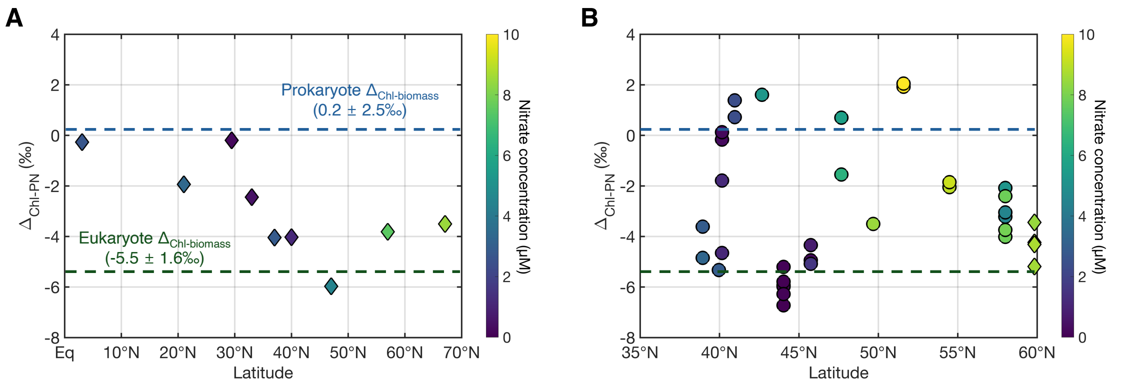

Fig. S2. Meridional distribution of Δ_{Chl-PN} from the North Atlantic cruise transects. (A) SYES, and (B) EN538. Nitrate concentration at sampling depth is color coded. Diamond symbols indicate the samples with nitrate data from the *World Ocean Atlas 2018* (15) due to the lack of bottle measurements. The two Δ_{Chl-PN} endmembers are delineated by colored dashed lines (green: eukaryotes, dark blue: prokaryotes).

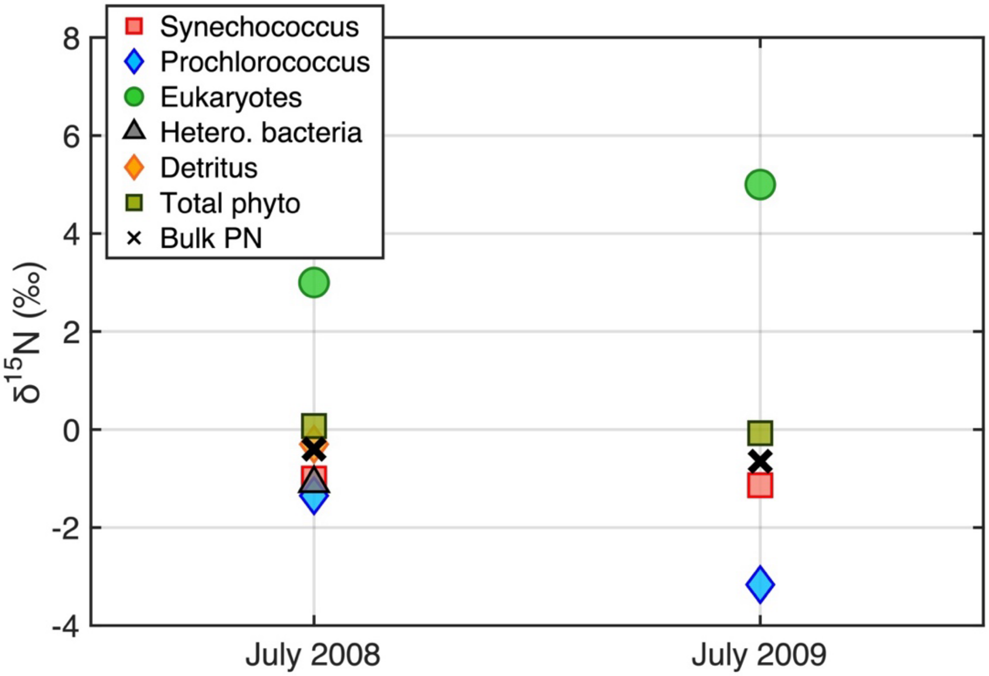

Fig. S3. $\delta^{15}N$ of flow cytometry sorted components of suspended particles from the Sargasso Sea in July 2008 and July 2009 (data from ref. 6). Total phytoplankton $\delta^{15}N$ (olive squares) is calculated with mass balance equation with the biomass concentrations and $\delta^{15}N$ values of Synechoccocus, Prochloroccocus, and eukaryotes ($\delta^{15}N_{Phytoplankton}$*$[N]_{Phytoplankton}$ = $\delta^{15}N_{Synechoccocus}$*$[N]_{Synechoccocus}$ + $\delta^{15}N_{Prochloroccocus}$*$[N]_{Prochloroccocus}$ + $\delta^{15}N_{Eukaryote}$*$[N]_{Eukaryote}$).

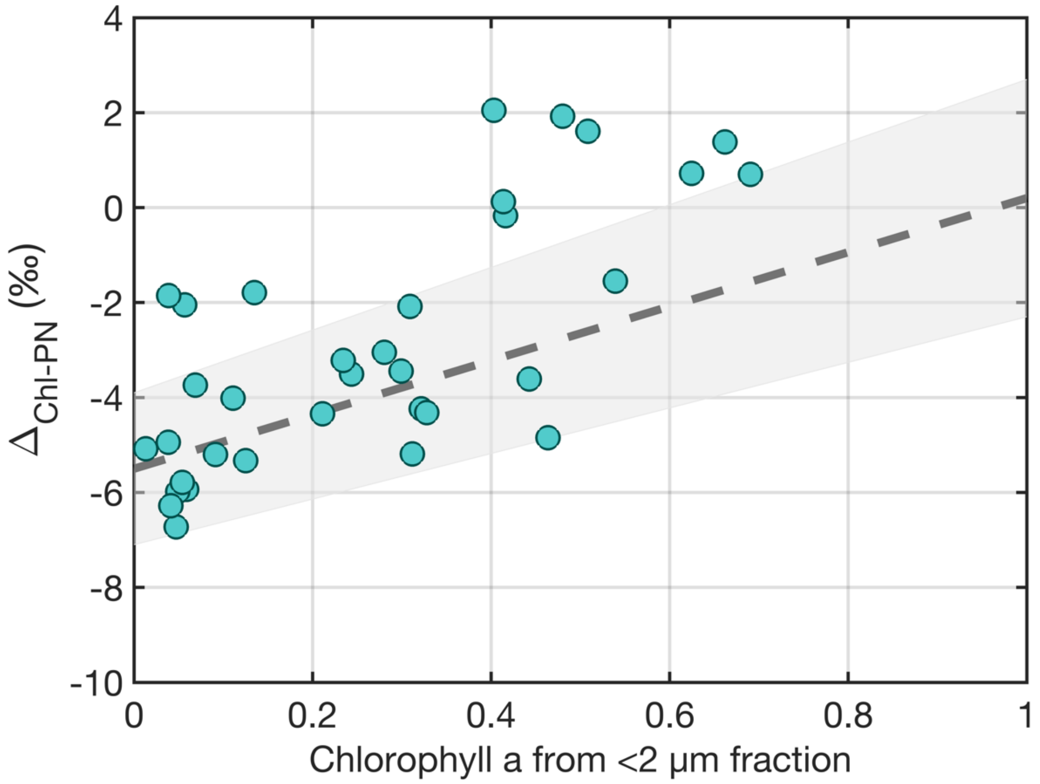

Fig. S4. Δ_{Chl-PN} values from the EN538 cruise data plotted versus fraction of chlorophyll a from smaller particles (<2 μm). The dashed grey line represents the estimated Δ_{Chl-PN} from the mixing line between eukaryotic algae (Δ_{Chl-PN} = −5.5 ± 1.6‰) and cyanobacteria (Δ_{Chl-PN} = 0.2 ± 2.5‰), under the assumption that particles greater/less than 2 μm derive from eukaryotic/prokaryotic phytoplankton. The grey shading that fills between the upper and lower Δ_{Chl-PN} estimation refers to the uncertainty in the Δ_{Chl-PN} endmembers.

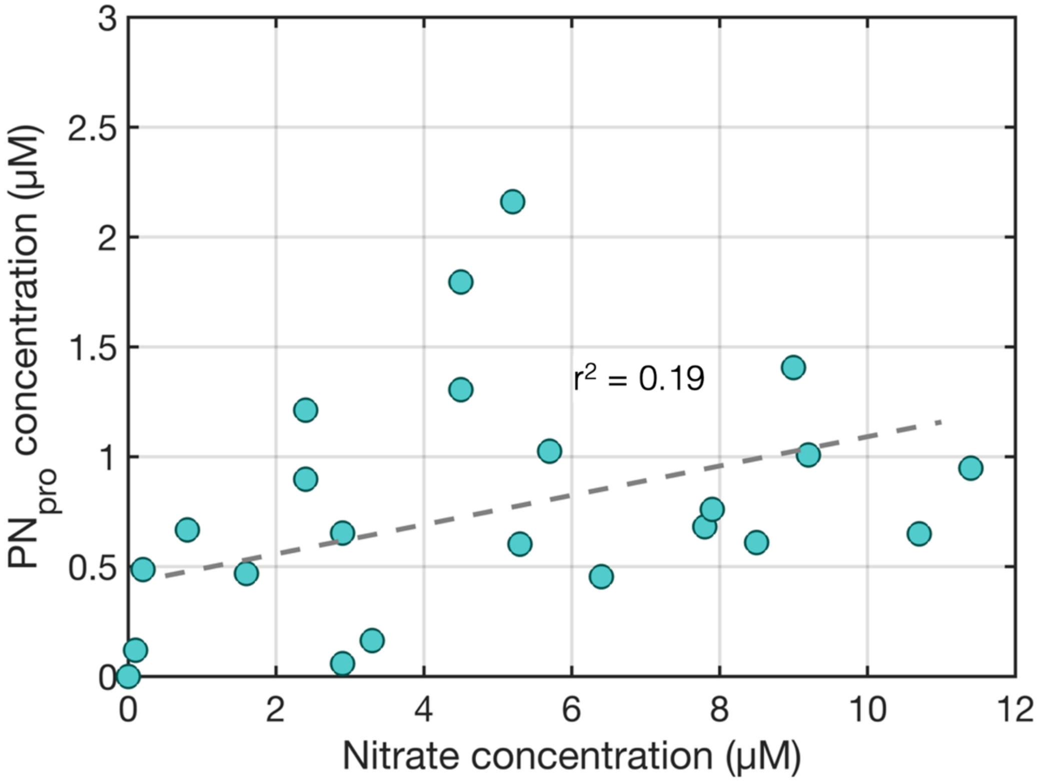

Fig. S5. Relationship between nitrate concentration and prokaryote-sourced suspended PN (PN_{pro}) from the EN538 cruise. PN_{pro} concentration is estimated by multiplying bulk suspended PN concentration with ($1 - f_{euk,PN}$). The linear regression curve is denoted as a grey dashed line.

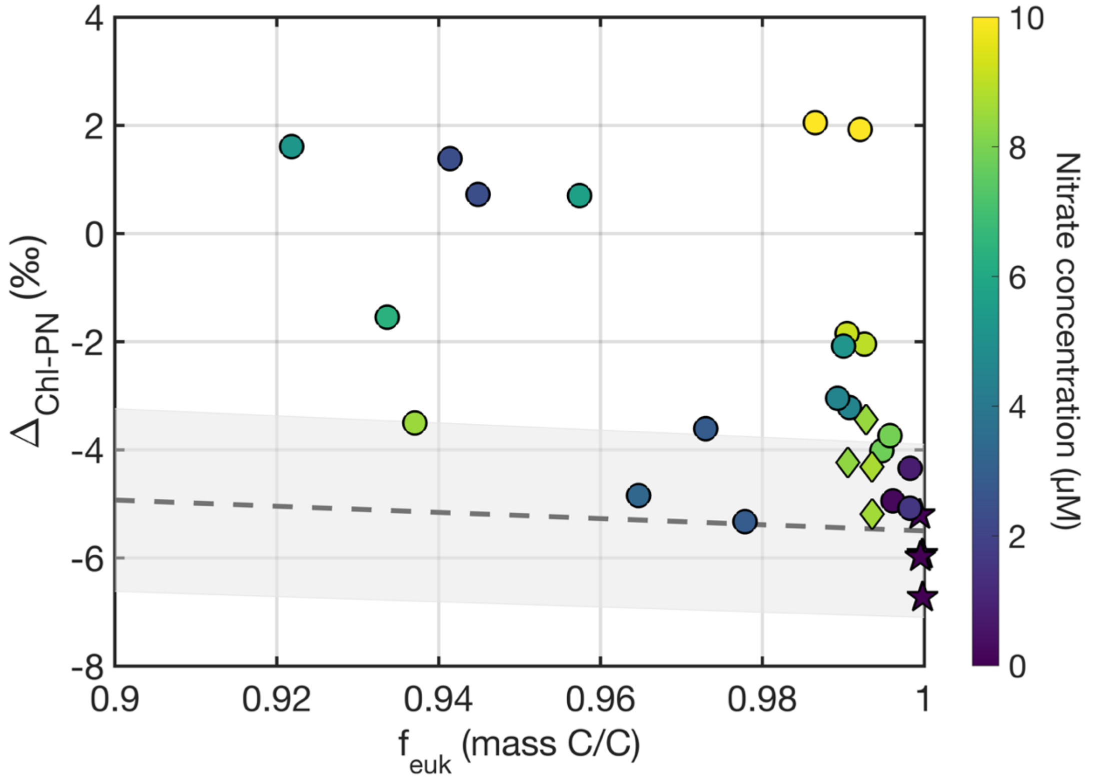

Fig. S6. Comparison between $\Delta_{Chl\text{-}PN}$ and $f_{euk,phyto}$ from the flow cytometry cell counting for EN538 samples. Nitrate concentration at sampling depths are color coded. Star symbols represent samples from shallow shelves over Grand Banks, and diamond symbols the samples with nitrate data from the *World Ocean Atlas 2018* (15). Circles indicate all other EN538 station measurements.

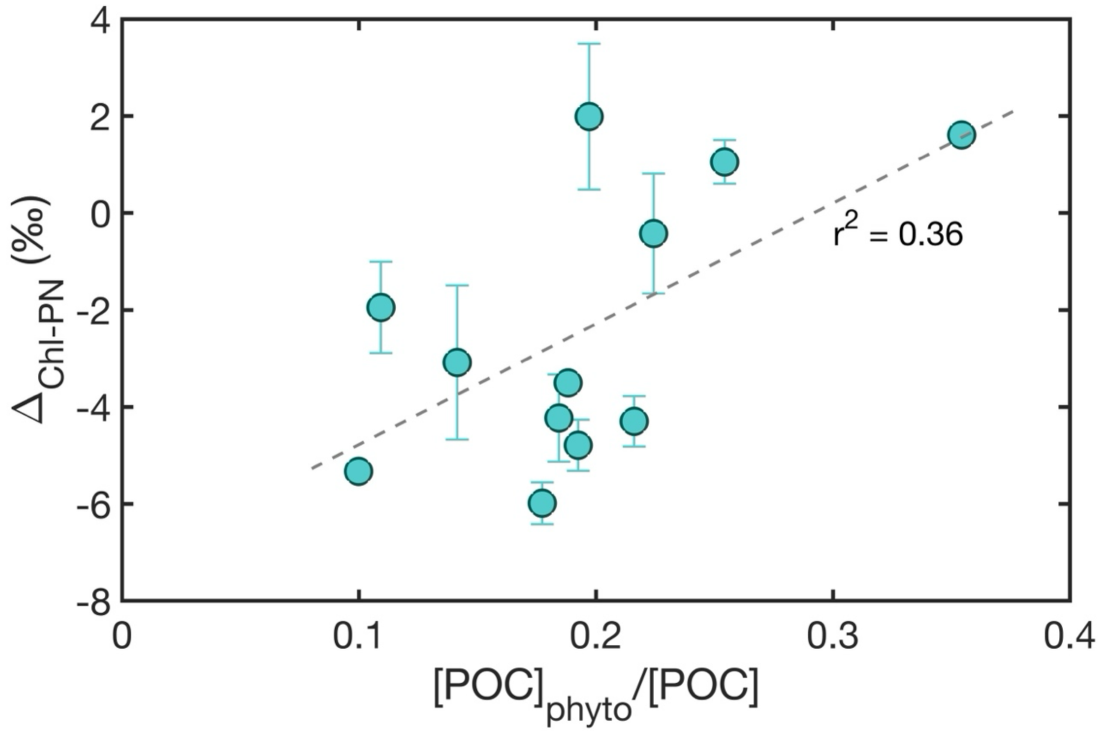

Fig. S7. Comparison of living phytoplankton biomass ($[POC]_{phyto}$) relative to total POC with $\Delta_{Chl\text{-}PN}$ at EN538 stations in the temperate North Atlantic (EN538). The grey dashed line indicates the regression.

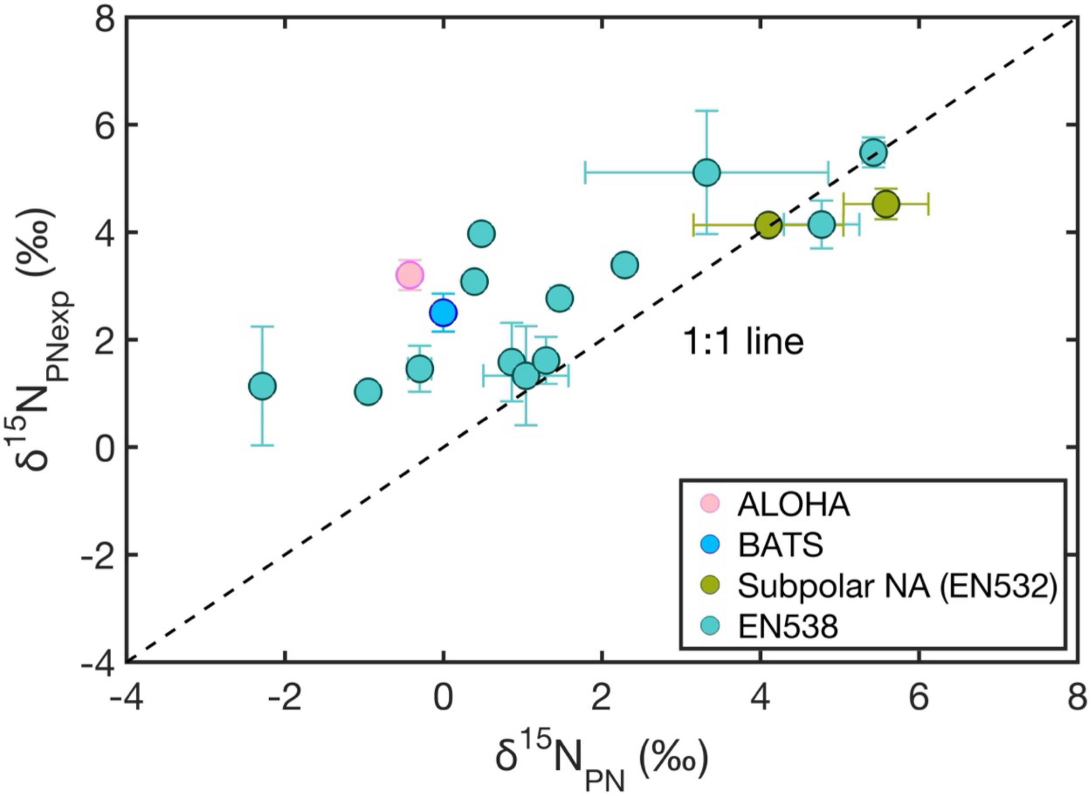

Fig. S8. Comparison of δ^{15}N between observed PN ($\delta^{15}N_{PN}$) and PN estimated from nitrate consumption ($\delta^{15}N_{PN,exp}$) from the subtropical gyres (pink: ALOHA, blue: BATS) and in the subpolar (olive: EN532) and temperate North Atlantic (cyan: EN538). The dashed grey line indicates the 1:1 relationship. Error bars show standard deviation (± 1σ). Here, $\delta^{15}N_{PN,exp}$ represents the δ^{15}N of the accumulated product estimated assuming Rayleigh-type isotope fractionation during nitrate assimilation: $\delta^{15}N_{PN,exp} = \delta^{15}N_{NO3} + f/(1-f)^* \ \varepsilon^*\ln(f)$, where f is the fraction of unutilized nitrate remaining ($[NO_3^-]/[NO_3^-]_{initial}$), $\delta^{15}N_{NO3}$ is δ^{15}N of subsurface nitrate, and ε is the isotope effect associated with nitrate assimilation.

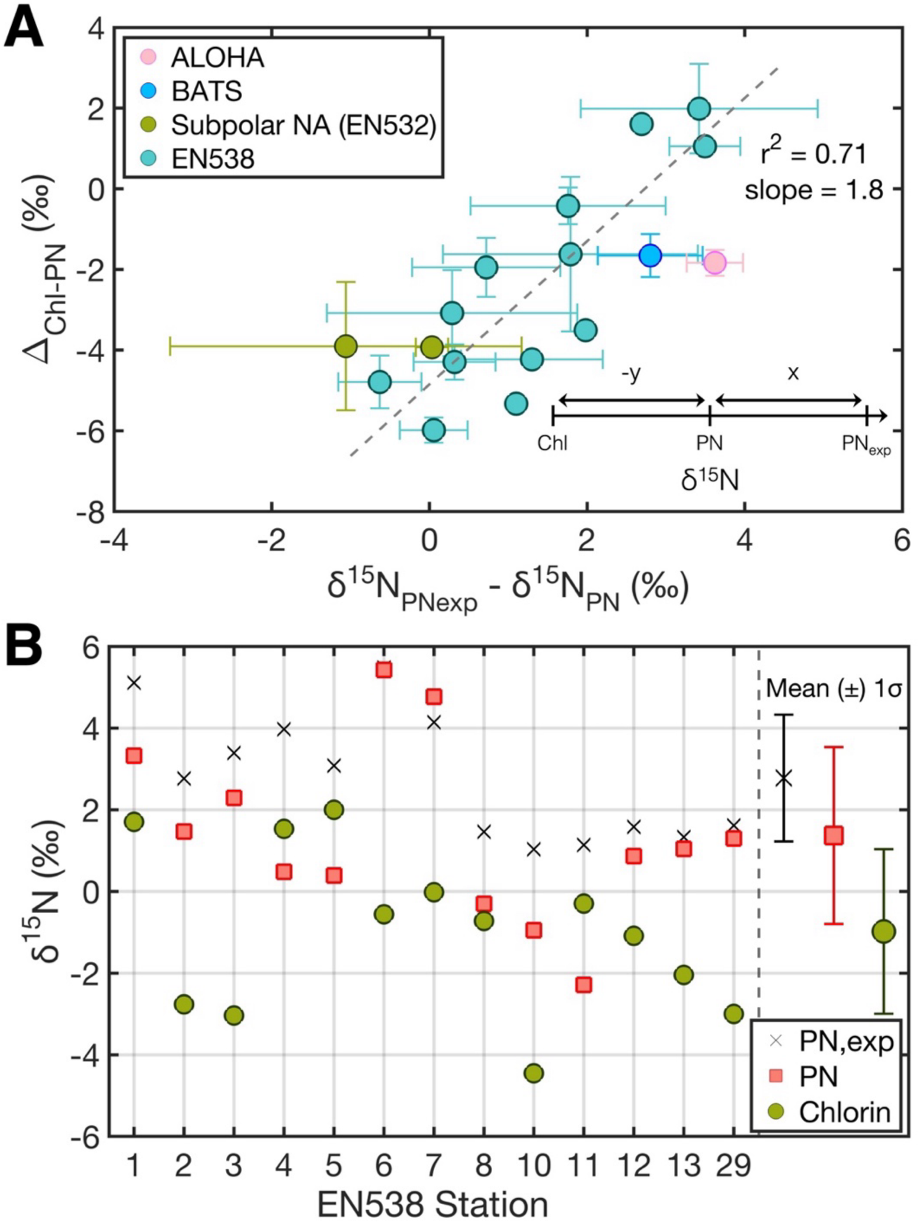

Fig. S9. (A) Relationship between the δ^{15}N offset ($\delta^{15}N_{PN,exp}$ – $\delta^{15}N_{PN}$) and $\Delta_{Chl\text{-}PN}$ across the subtropical gyres (pink: ALOHA, blue: BATS) and the subpolar (olive: EN532) and temperate North Atlantic (cyan: EN538). (B) δ^{15}N values at each EN538 station (black crosses: $\delta^{15}N_{PN,exp}$, red squares: $\delta^{15}N_{PN}$, olive circles: $\delta^{15}N_{Chl}$). $\delta^{15}N_{PN,exp}$ values were estimated from nitrate consumption and nitrate δ^{15}N. In (A), the grey dashed line shows the regression for EN538 data, with slope and correlation coefficient (r^2 = 0.71) given in the panel. For clarity, the lower-right inset of (A) shows the δ^{15}N distributions of PN_{exp}, PN, and chlorin, as well as the variables plotted on the x- and y-axes. (B) shows informative station-to-station variations with regard to the importance of nitrate consumption and eukaryote dominance of the PN pool. These variations also indicate that the correlation (A) is not simply due to autocorrelation arising from the inclusion of $\delta^{15}N_{PN}$ in both the x and y axes of (A). In the right panel of (B), symbols and error bars indicate the station mean and standard error (± 1σ). Metadata for each station are provided in Table S3.

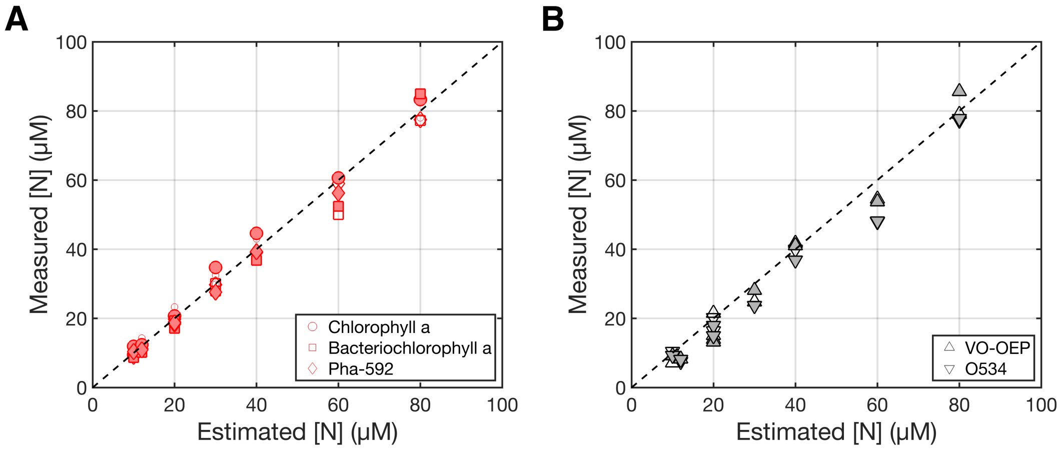

Fig. S10. Oxidation yields of chlorin compounds using persulfate oxidizing reagent (POR). (A: chlorin compounds, B: porphyrin). Samples subjected to 6-hour UV treatment are denoted by the closed symbols while open symbols indicate samples not exposed to UV. Dashed lines signify a 100% yield reference.

Table S1. $\delta^{15}N_{PN}$ and $\delta^{15}N_{Chl}$ data from previous studies and their comparison with $f_{euk,phyto}$. (numbers in parenthesis represent references).

Sampling location	$\delta^{15}N_{PN}$	$\delta^{15}N_{Chl}$	$\delta^{15}N_{PN}$	$f_{euk,PN}$	$f_{euk,phyto}$
Arabian Sea	9.1‰	5.57‰	-3.53‰	0.65 (11)	0.48-0.8 (16)
Southern Ocean	6.7‰	2.52‰	-4.18‰	0.77 (11)	>0.98 (17)
Eastern Equatorial Pacific	6.1‰	1.77‰	-4.33‰	0.79 (11)	0.35-0.86 (18)
Eastern Mediterranean (Ionian Sea)	-0.63‰	-6.38‰	-5.75‰	1.00 (12)	0.84-0.9 (19)
Western Mediterranean (Alboran Sea)	2.22‰	-2.52‰	-4.74‰	0.87 (12)	0.84-0.98 (20)
Northwest Pacific subtropical gyre	2.0‰	0‰	-2.0‰	0.39 (13)	0.45 ± 0.16 (21)
Subarctic North Pacific	0.6‰	-4.8‰	-5.4‰	0.98 (13)	0.96 (22)

Table S2. Calculated $[POC]_{Phyto}/[POC]$ over the sampling stations. For the BATS station, the flow cytometry sorted cell biomass and suspended PN data from ref. 6 are used. PN-to-POC conversion includes uncertainty from latitudinal elemental ratio variation (14).

Sampling station	$[POC]_{Phyto}/[POC]$
BATS (July 2008, July 2009)[6]	0.46 ± 0.18
Oregon Coast	0.11 ± 0.08
EN538	0.19 ± 0.12
SYES 2019-2021	0.34 ± 0.20

Table S3. Metadata for filter samples analyzed for $\delta^{15}N$ of bulk PN and chlorin.

Sampling Stations	Date	Latitude (°N)	Longitude (°E)	Depth (m)	Temperature (°C)	Salinity (psu)	$[NO_3^-]$ (μM)	$\delta^{15}N_{PN}$ (‰)	$\delta^{15}N_{Chl}$ (‰)	[PN] (μM)	Pro. (nM C)	Syn. (nM C)	Pico. (nM C)	Nano. (nM C)	Total Phyto. (nM C)	Chl (>20 μm) (ng L^{-1})	Chl (20-2 μm) (ng L^{-1})	Chl (<2 μm) (ng L^{-1})	Total Chl (ng L^{-1})
EN532	9/3/13	54.02	-20.44	15	13.8	35.3	0.0	4.0	0.3	1.8	0	255	990	4303	6076	22	386	739	1147
EN532	9/3/13	54.02	-20.44	25	13.8	35.3	2.1	4.2	0.1	1.8	0	262	1044	4088	6020	21	407	477	905
EN532	9/8/13	50.00	-20.00	15	14.9	35.1	0.0	5.4	2.4	3.4	0	568	293	6621	7287	9	405	622	1256
EN532	9/8/13	50.00	-20.00	20	14.9	35.1	0.0	5.7	2.9	2.4	0	420	454	3872	5275				
EN532	9/8/13	50.00	-20.00	35	14.9	35.1	0.0	5.9	2.0	2.1	0	305	469	2227	4064				
EN532	9/8/13	50.00	-20.00	45	13.9	35.3	1.6	5.3	-0.6	1.3	0	120	281	908	2033				578
EN538 (Station 1)	4/30/14	40.16	-69.31	10	20.4	36.3	0.3	2.9	2.7	1.2						302	102	288	692
EN538 (Station 1)	4/30/14	40.16	-69.31	20	20.4	36.3	0.2	2.0	2.2	1.0						318	103	297	718
EN538 (Station 1)	4/30/14	40.16	-69.31	40	13.7	34.9	1.2	3.7	1.9	1.8						1368	142	235	1744
EN538 (Station 1)	4/30/14	40.16	-69.31	50	12.8	34.6	1.1	4.7	0.1	2.6									
EN538 (Station 2)	5/1/14	38.94	-65.82	30	16.5	36.0	2.9	1.5	-2.1	2.0	0	66	94	2285	2445	353	231	463	1046
EN538 (Station 2)	5/1/14	38.94	-65.82	50	16.5	36.1	3.3	1.4	-3.4	1.4	0	61	84	1556	1700	305	213	448	966
EN538 (Station 3)	5/2/14	39.96	-60.88	30	15.9	36.0	2.9	2.3	-3.0	1.9	0	28	44	1192	1264	1097	136	176	1408
EN538 (Station 4)	5/3/14	40.96	-56.93	20	17.0	36.2	2.4	0.5	1.2	1.2	0	101	89	1642	1832	56	175	386	618
EN538 (Station 4)	5/3/14	40.96	-56.93	50	17.0	36.2	2.4	0.5	1.8	0.9	0	97	90	1468	1655	40	149	370	559
EN538 (Station 5)	5/4/14	42.67	-52.98	30	14.4	35.7	5.3	0.4	2.0	0.6	0	110	44	1253	1407	25	142	182	358
EN538 (Station 6)	5/5/14	44.03	-49.72	8	1.2	32.8	0.0	5.4	-0.5	2.3	0	72	23	4085	4109	1457	92	97	1646
EN538 (Station 6)	5/5/14	44.03	-49.72	15	1.1	32.8	0.0	5.9	-0.9	3.8	0	85	24	5052	5077	1784	91	93	1968
EN538 (Station 6)	5/5/14	44.03	-49.72	20	1.1	32.8	0.0	5.6	-0.4	2.8	0	53	28	2101	2130	2246	123	122	2490
EN538 (Station 6)	5/5/14	44.03	-49.72	25	1.1	32.8	0.0	5.3	-0.5	2.4						2268	143	138	2549
EN538 (Station 6)	5/5/14	44.03	-49.72	35	1.2	32.8	0.1	5.1	-0.1	2.3	0	63	21	1805	1827	2090	229	233	2552
EN538 (Station 6)	5/5/14	44.03	-49.72	45	1.2	32.8	0.1	5.3	-1.0	2.3						2247	151	103	2501
EN538 (Station 7)	5/6/14	45.75	-45.14	20	4.7	33.3	0.8	4.3	0.0	3.3	0	21	104	11287	11411	194	1140	357	1691
EN538 (Station 7)	5/6/14	45.75	-45.14	35	1.8	33.6	0.2	5.2	0.3	5.0	0	2	18	757	778	3256	208	138	3602

EN538 (Station 7)	5/6/14	45.75	-45.14	50	1.7	33.6	1.6	4.8	-0.3	6.4	0	2	21	1107	1130	6328	202	87	6617
EN538 (Station 8)	5/7/14	47.67	-39.74	20	13.6	35.7	5.7	-0.6	0.1	1.0	0	85	136	1775	1996	46	137	408	591
EN538 (Station 8)	5/7/14	47.67	-39.74	50	13.0	35.6	6.4	0.0	-1.5	0.7	0	44	47	572	663	46	67	131	243
EN538 (Station 10)	5/9/14	49.68	-33.82	20	10.9	35.3	8.5	-0.9	-4.5	1.7	0	137	78	1947	2161	692	155	273	1121
EN538 (Station 11)	5/10/14	51.59	-28.17	20	9.0	35.1	11.4	-1.5	0.4	1.5	0	18	68	2186	2272	20	204	206	429
EN538 (Station 11)	5/10/14	51.59	-28.17	40	8.7	35.1	10.7	-3.1	-1.0	1.0	0	14	36	991	1041	45	189	158	392
EN538 (Station 12)	5/11/14	54.48	-24.62	20	8.5	35.1	9.0	1.4	-0.7	2.3	0	11	16	1463	1490	965	131	66	1162
EN538 (Station 12)	5/11/14	54.48	-24.62	40	8.5	35.1	9.2	0.3	-1.5	1.6	0	12	21	1226	1259	1017	145	47	1209
EN538 (Station 13)	5/12/14	58.00	-20.00	10	10.4	35.3	4.5	2.0	-1.2	3.3	0	43	38	4577	4658	289	224	157	670
EN538 (Station 13)	5/12/14	58.00	-20.00	20	10.3	35.3	4.5	2.2	-0.8	4.2	0	49	41	4485	4575	222	245	182	649
EN538 (Station 13)	5/12/14	58.00	-20.00	25	10.3	35.3	5.2	1.3	-0.8	3.6	0	44	42	4323	4409	268	311	259	838
EN538 (Station 13)	5/12/14	58.00	-20.00	30	10.0	35.3	7.8	0.1	-2.3	2.4									
EN538 (Station 13)	5/12/14	58.00	-20.00	37	9.9	35.3	7.8	0.4	-3.6	2.6	0	5	18	1123	1147	599	129	91	820
EN538 (Station 13)	5/12/14	58.00	-20.00	45	9.7	35.3	7.9	0.2	-3.5	2.5	0	5	15	1161	1181	729	181	67	977
EN538 (Station 29)	5/17/14	59.83	-21.59	5			8.5	0.8	-2.7	1.7	0	24	72	3238	3334	175	352	225	752
EN538 (Station 29)	5/17/14	59.83	-21.59	10			8.6	1.8	-3.4	2.8	0	25	31	3831	3887	310	190	226	725
EN538 (Station 29)	5/17/14	59.83	-21.59	20			8.3	1.3	-2.9	2.4	0	20	52	2048	2120	323	223	259	805
EN538 (Station 29)	5/17/14	59.83	-21.59	30			8.7	1.3	-3.0	2.9	0	26	31	4131	4189	301	201	245	747
BATS (n=6)	6/6/19	31.83	-64.17	5				-0.1 ± 0.1	-1.5 ± 0.2	0.3									
ALOHA (n=4)	6/22/19 – 6/23/19	22.75	-158.00	5	26.4 – 27.0	35.1 – 35.3		-0.4 ± 0.3	-2.2 ± 0.2	0.3									
ALOHA	6/21/19	22.75	-158.00	25				-1.0 ± 0.3	-1.6	0.17*									
ALOHA	6/21/19	22.75	-158.00	45				-1.0 ± 0.3	-2.6	0.20*									
ALOHA	6/20/19	22.71	-157.93	75				-0.3 ± 0.4	-1.4	0.19*									
ALOHA	6/16/19	22.76	-158.07	100				-0.6 ± 0.8		0.15*									
ALOHA	6/17/19	22.75	-158.00	105				1.4 ± 0.2	-0.6										

ALOHA	6/20/19	22.71	-157.93	125				0.4 ± 0.1	-2.2	0.17*									
ALOHA	6/19/19	22.67	-158.01	140				1.2 ± 0.6	-0.8										
ALOHA	6/17/19	22.77	-157.92	150				1.6 ± 0.2		0.10*									
ALOHA	6/22/19	22.83	-158.02	175				5.6	0.8	0.04*									
ALOHA	6/17/19	22.77	-158.01	175				3.8											
ALOHA	6/22/19	22.83	-158.02	225				4.7 ± 0.9	0.7	0.03*									
ALOHA	6/17/19	22.77	-157.92	300				4.4 ± 0.3		0.01*									
ETNP	4/12/18	10.00	-111.00	5	28.3	33.8	0.1	6.5	0.0	0.5									
ETNP	4/12/18	15.77	-103.00	5	28.3	34.0	0.1	6.3	-0.3	0.0									
ETNP	4/2/18	17.68	-101.65	5	28.0	34.6	0.3	8.5	-0.8	0.1									
SYES2019	8/14/19	40.00	-20.00	30	18.7	35.9	1.0	4.2	0.1	0.1			195	127	322				
SYES2020	7/12/20	67.08	-5.97	36	6.9	35.0	7.2	-2.9 ± 0.3	-6.4		1	1425	8944	4406	14776				
SYES2020	7/12/20	67.08	-5.97	143				5.0 ± 0.3	-3.4										
SYES2020	7/22/20	56.98	-19.99	39	12.5	35.3	5.0	2.4 ± 0.5	-1.4	1.3	4	1168	1152	2054	4378				
SYES2020	7/22/20	56.98	-19.99	201				5.1 ± 0.2	-3.2										
SYES2020	8/10/20	47.00	-20.00	45	15.6	35.7	4.5	3.4 ± 0.5	-2.5	2.0		15	59	698	772				
SYES2020	8/10/20	47.00	-20.00	173				6.1 ± 0.1	-2.5										
SYES2020	8/22/20	37.01	-20.98	103	17.1	36.3	2.9	1.1 ± 0.3	-2.9		378	3	363	2786	3529				
SYES2020	8/22/20	37.01	-20.98	208				5.7 ± 0.2	-0.1										
SYES2020	9/19/20	33.00	-22.00	85	20.3	36.6	0.5	-0.5 ± 0.3	-2.9	0.5	269	1	180	1000	1450				
SYES2020	9/19/20	33.00	-22.00	153				2.4 ± 0.5	-2.9										
SYES2020	11/8/20	29.45	-15.05	58	22.2	36.8	0.2	-1.8 ± 0.3	-2.0	0.2									
SYES2020	11/8/20	29.45	-15.05	210				5.4 ± 0.6	0.6										
SYES2021	4/16/21	21.02	-20.95	48	20.0	36.5	3.4	2.9 ± 1.0	1.0	0.8	1	493	610	824	1927				
SYES2021	5/17/21	3.07	-21.94	49	28.8	35.7	2.7	1.2 ± 0.3	0.9	0.4	1	104	534	256	895				
SYES2021	5/17/21	3.07	-21.94	124				4.8	-0.2										

PUPCYCLE II	5/30/23	43.04	-124.55	5			12.7	5.1	-1.5	0.9		27	177	602	807				
PUPCYCLE II	5/31/23	42.94	-124.42	5			12.5	4.9	-1.6	3.9		61	399	1274	1734				
PUPCYCLE II	6/2/23	42.68	-124.67	5			4.3	7.2	1.0			17	106	325	448				
PUPCYCLE II	6/3/23	42.41	-124.67	5			17.5	5.3	-1.3			2	44	93	139				
PUPCYCLE II	6/6/23	39.22	-124.30	5			6.5	4.5	-2.2	3.1		5	379	1811	2195				
PUPCYCLE II	6/7/23	39.22	-124.23	5			4.8	3.9	-2.9	3.0		39	243	743	1025				
PUPCYCLE II	6/8/23	39.05	-123.90	5			18.7	4.6	-1.6	4.0		27	282	1420	1810				
PUPCYCLE II	6/8/23	37.92	-124.01	5			11.9	7.1	0.7	1.3		11	873	1421	2107				
**Some Station ALOHA PN data from DOI: 10.26008/1912/bco-dmo.853048.1 (23)*																			

Table S4. Types of glass fiber filters used for surface particle analyses and corresponding $\delta^{15}N$ data. A tandem configuration of 1.2 μm and 0.3 μm pore-size filters was utilized for Station ALOHA samples and the majority of the BATS station samples.

Sampling location	Filter type	Pore size	$\delta^{15}N_{PN}$	$\delta^{15}N_{Chl}$
ALOHA (pre-filtered with 51 μm mesh)	GF/C 150 mm	1.2 μm (in series)	-0.4 ± 0.3‰	-2.2 ± 0.2‰
	GF-75 142 mm	0.3 μm (in series)	-0.1 ± 0.3‰	
ALOHA subsurface (pre-filtered with 51 μm mesh)	GF/C 47 mm	1.2 μm	−1.0 ~ +4.7‰	−2.6 ~ +0.8‰
	GF/F 142 mm	0.7 μm		
BATS (pre-filtered with 51 μm mesh)	GF/C 150 mm	1.2 μm (in series)	0.1 ± 0.3‰	-1.5 ± 0.4‰
	GF-75 142 mm	0.3 μm (in series)	0.4 ± 0.3‰	
	GF-75 142 mm	0.3 μm	-0.1 ± 0.1‰	-1.5 ± 0.2‰
ETNP	GF-75 142mm	0.3 μm	7.1 ± 1.2‰	−0.4 ± 0.4‰
Oregon Coast (PUPCYCLE II)	GF-75 142 mm	0.3 μm	5.3 ± 1.2‰	−1.2 ± 1.3‰
Subpolar N. Atl. (EN532)	GF-75 47 mm	0.3 μm	5.4 ± 1.2‰	1.2 ± 1.4‰
Eastern N. Atl. (SYES)	GF-75 142 mm	0.3 μm	−2.9 ~ +4.2‰	−6.4 ~ +1.0‰
			+2.3 ~ +6.1‰ (subsurface)	−3.4 ~ +0.1‰ (subsurface)
N. Atl. (EN538)	GF-75 47 mm	0.3 μm	−3.0 ~ +5.9‰	−4.5 ~ +2.7‰

Table S5. N isotopic comparison of chlorin standards measured using the denitrifier method with and without UV treatment, and the corresponding EA-IRMS measurements.

Compound	No UV treatment	UV treatment (6 hours)	EA-IRMS
Chlorophyll a	10.8 ± 0.2‰	10.6 ± 0.3‰	
Bacteriochlorophyll a	-12.5 ± 0.2‰	-12.4 ± 0.5‰	
PHA-592	-1.2 ± 0.1‰	-1.2 ± 0.1‰	-1.4 ± 0.1‰
Vanadyl Octaethylporphine (VO-OEP)	-7.4 ± 0.1‰	-7.4 ± 0.2‰	-7.8 ± 0.1‰
Octaethylporphine (O534)	-6.6 ± 0.1‰	-6.8 ± 0.2‰	-6.7 ± 0.1‰

SI References

1. J. P. Sachs, D. J. Repeta, Oligotrophy and nitrogen fixation during eastern Mediterranean sapropel events. *Science* 286, 2485–2488 (1999).
2. J. M. Hayes, K. H. Freeman, B. N. Popp, C. H. Hoham, Compound-specific isotopic analyses: A novel tool for reconstruction of ancient biogeochemical processes. *Org. Geochem.* 16, 1115–1128 (1990).
3. J. Tyler *et al.,* Tracking aquatic change using chlorine-specific carbon and nitrogen isotopes: The last glacial-interglacial transition at Lake Suigetsu, Japan. *Geochem. Geophys. Geosyst.* 11, 1–19 (2010).
4. N. Kawasaki, R. Sohrin, H. Ogawa, T. Nagata, R. Benner, Bacterial carbon content and the living and detrital bacterial contributions to suspended particulate organic carbon in the North Pacific Ocean. *Aquat. Microb. Ecol.* 62, 165–176 (2011).
5. A. Andersson, A. Rudehall, Proportion of plankton biomass in particulate organic carbon in the northern Baltic Sea. *Mar. Ecol. Prog. Ser.*95, 133–139 (1993).
6. S. E. Fawcett, M. W. Lomas, J. R. Casey, B. B. Ward, D. M. Sigman, Assimilation of upwelled nitrate by small eukaryotes in the Sargasso Sea. *Nat. Geosci.* 4, 717–722
7. A. Gutiérrez-Rodríguez, M. Décima, B. N. Popp, M. R. Landry, Isotopic invisibility of protozoan trophic steps in marine food webs. *Limnol. Oceanogr.* 59, 1590–1598 (2014).
8. Möbius, J, Isotope fractionation during nitrogen remineralization (ammonification): Implications for nitrogen isotope biogeochemistry. *Geochim. Cosmochim. Ac.* 105, 422–432 (2013).
9. A. N. Knapp, D. M. Sigman, F. Lipschultz, A. B. Kustka, D. G. Capone, Interbasin isotopic correspondence between upper-ocean bulk DON and subsurface nitrate and its implications for marine nitrogen cycling. *Global. Biogeochem. Cy.* 25, 1–14 (2011).
10. S. E. Fawcett et al., Unconditional nutrient niches for open ocean phytoplankton. Abstract presented at *2026 Ocean Sciences Meeting*, AGU, Glasgow, Scotland (2026).
11. J. P. Sachs, D. J. Repeta, R. Goericke, Nitrogen and carbon isotopic ratios of chlorophyll from marine phytoplankton. *Geochim. Cosmochim. Ac.* 63, 1431–1441 (1999).
12. S. Pantoja, D. J. Repeta, J. P. Sachs, D. M. Sigman, Stable isotope constraints on the nitrogen cycle of the Mediterranean Sea water column. *Deep. Sea. Res. Pt I.* 49(9), 1609–1621 (2002).
13. C. Yoshikawa *et al.,* Insight into nitrous oxide production processes in the western North Pacific based on a marine ecosystem isotopomer model. *J. Oceanogr.* 72(3), 491–508 (2016).
14. T. Tanioka et al., Global patterns and predictors of C:N:P in marine ecosystem. *Commun. Earth Environ.* 3, 271 (2022).
15. H. Garcia *et al.,* “World Ocean Atlas 2018 volume 4: Dissolved inorganic nutrients (phosphate, nitrate and nitrate + nitrite, silicate)” (NOAA Atlas NESDIS 84, 2019); URL. [https://data.nodc.noaa.gov/woa/WOA18/DOC/woa18_vol4.pdf]
16. A. Shalapyonok, R. J. Olson, L. S. Shalapyonok, Arabian Sea phytoplankton during Southwest and Northeast Monsoons 1995: composition, size structure and biomass from individual cell properties measured by flow cytometry. *Deep. Sea. Res. Pt II.* 48(6-7), 1231-1261 (2001).
17. J. J. Viljoen *et al.,* Links between the phytoplankton community composition and trace metal distribution in summer surface waters of the Atlantic Southern Ocean. *Front. Mar. Sci.* 6 (2019).
18. A. G. Taylor, M. R. Landry, K. E. Selph, E. J. Yang, Biomass, size structure and depth distributions of the microbial community in the eastern equatorial Pacific. *Deep. Sea. Res. Pt II.* 58(3-4), 342-357 (2011). doi: 10.1016/j.dsr2.2010.08.017
19. R. Casotti *et al.,* Composition and dynamics of the phytoplankton of the Ionian Sea (eastern Mediterranean). *J. Geophys. Res. Oceans*. 108(C9) (2003).
20. C. Videau, A. Sournia, L. Prieur, M. Fiala, Phytoplankton and primary production characteristics at selected sites in the geostrophic Almeria-Oran front system (SW Mediterranean Sea). *J. Mar. Syst.* 5, 235–250 (1994).

21. M. Girault *et al.,* Distribution of ultraphytoplankton in the western part of the North Pacific subtropical gyre during a strong La Niña condition: relationship with the hydrological conditions. *Biogeosciences*, 10(9), 5947–5965 (2013).
22. T. Fujiki *et al.,* Seasonal cycle of phytoplankton community structure and photophysiological state in the western subarctic gyre of the North Pacific. *Limnol. Oceanogr.* 59(3), 887–900 (2014).
23. Black, E., Maloney, A., Subhas, A. V., Kenyon, J., Church, M. J., White, A. E., Goetze, E., Ferron, S. (2021). Suspended particles from in situ pumps on R/V Kilo Moana cruise KM1910 in June 2019. Biological and Chemical Oceanography Data Management Office (BCO-DMO). Version 1 (2021-06-04). doi:10.26008/1912/bco-dmo.853048.1 [March 15 2026]
